# Phenazine Derivatives with Anti-Inflammatory Activity from the Deep-Sea Sediment-Derived Yeast-Like Fungus *Cystobasidium laryngis* IV17-028

**DOI:** 10.3390/md17080482

**Published:** 2019-08-19

**Authors:** Hwa-Sun Lee, Jong Soon Kang, Byeoung-Kyu Choi, Hyi-Seung Lee, Yeon-Ju Lee, Jihoon Lee, Hee Jae Shin

**Affiliations:** 1 Marine Natural Products Chemistry Laboratory, Korea Institute of Ocean Science Technology, 385 Haeyang-ro, Yeongdo-gu, Busan 49111, Korea; 2 Laboratory Animal Resource Center, Korea Research Institute of Bioscience and Biotechnology, 30 Yeongudangi-ro, Ochang-eup, Cheongwon-gu, Cheongju 28116, Korea

**Keywords:** deep-sea sediment, marine yeast-like fungi, *Cystobasidium laryngis*, phenazine derivatives, saphenic acid, anti-inflammatory activity

## Abstract

Three new phenazine derivatives (**1**–**3**), along with known compounds (**4**–**7**) of saphenic acid derivatives, were isolated from a deep-sea sediment-derived yeast-like fungus *Cystobasidium larynigs* collected from the Indian Ocean. The structures of the new compounds (**1**–**3**) were determined by analysis of spectroscopic data, semi-synthesis and comparison of optical rotation values. All the isolated compounds (**1**–**7**), except for **2**, showed nitric oxide (NO) production inhibitory effect against lipopolysaccharide (LPS)-induced murine macrophage RAW 264.7 cells without cytotoxicity at concentrations up to 30 μg/mL. This is the first report on the yeast-like fungus *Cystobasidium laryngis* producing phenazines and anti-inflammatory activity of **1**–**7** including saphenic acid (**4**).

## 1. Introduction

Natural products and their derivatives have been recognized as an attractive source of drug discovery [1]. In particular, microorganisms from the marine environment are a rich source of structurally unique bioactive metabolites and have produced a number of drug candidates [2]. Among them, marine microbes from the deep-sea are a relatively untapped reservoir of metabolites with structural and biological diversity waiting to be discovered because of lack of technology and the difficulty of collecting samples [3].

In our continuing search for bioactive substances from deep-sea microorganisms, we encountered a rare marine-derived yeast-like fungus *Cystobasidium laryngis* isolated from a deep-sea sediment sample collected from the Indian Ocean Ridge in 2017. This yeast-like fungus was designated as IV17-028 and cultivated in a large scale. Phenazine compounds (**1**–**7**) including saphenic acid (**4**) were isolated from the extract of the culture broth by chromatographic methods and recrystallization. Phenazines are a large group of redox-active secondary metabolites produced by various bacteria (*Streptomyces* and *Pseudomonas*) and some archea [4]. Phenazines share a diabenzo annulated pyrazine core structure and have a broad spectrum of biological activities, such as anti-microbial, anti-cancer, cancer chemopreventive, anti-malarial and anti-parasitic [5]. Among them, saphenic acid containing phenazine core is a common pharmacophore for antibiotics and antitumor agents including saphenamycin and phenazostatins which were isolated from *Streptomyces* sp. [6].

**Figure 1:**
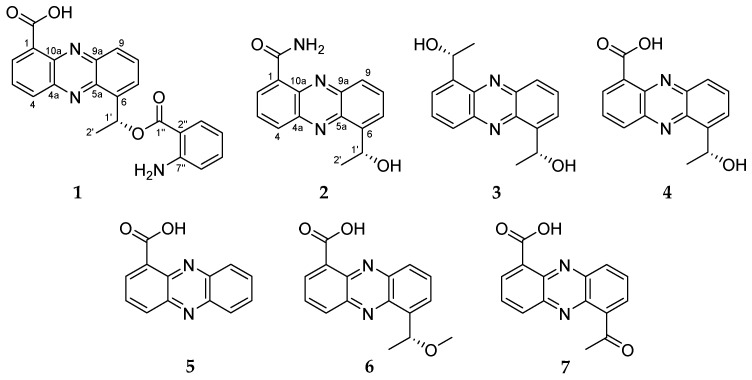
The structures of compounds **1**–**7**.

Here we report the isolation of *Cystobasidium laryngis* IV17-028, fermentation, purification of seven phenazine derivatives (**1**–**7**), structure determination of three new compounds **1**–**3** and biological activity of all the isolated compounds **1**–**7** (Figure 1). This is the first report on the phenazine derivatives from the marine yeast-like fungus *Cystobasidium laryngis* and their anti-inflammatory activity.

## 2. Results and Discussion

**Figure 2:**
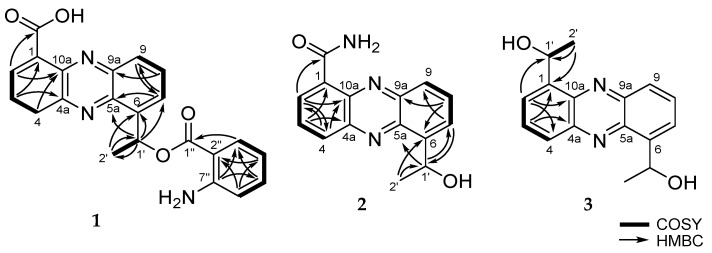
The Key COSY and HMBC correlations of **1**–**3**.

Compound **1** was isolated as a yellowish amorphous solid. The molecular formula of **1** was deduced to be C_22_H_17_N_3_O_4_ from the [M + H]^+^ peak at *m*/*z* 388.1296 (calcd. for 388.1297) in the HRESIMS, which required 16 degrees of unsaturation. The ^1^H and HSQC spectra of **1** showed ten aromatic proton signals at δ_H_ 8.90 (1H, d, *J* = 7.4 Hz), 8.70 (1H, d, *J* = 8.6 Hz), 8.23 (1H, d, *J* = 7.7 Hz), 8.15 (1H, t, *J* = 7.4 Hz), 7.99 (1H, overlapped), 7.98 (1H, overlapped), 7.89 (1H, d, *J* = 7.6 Hz), 7.03 (1H, t, *J* = 8.0 Hz), 6.49 (1H, t, *J* = 7.6 Hz) and 6.42 (1H, d, *J* = 8.0 Hz), one methine signal at δ_H_ 6.06 (1H, q, *J* = 6.4 Hz) and one methyl signal at δ_H_ 1.81 (3H, d, *J* = 6.4 Hz) (Figure S1). Moreover, an exchangeable proton signal of a carboxyl group at δ_H_ 15.60 (1H, s) was detected on the ^1^H NMR spectrum using CDCl_3_ (Table 1) (Figure S2). The ^13^C (Figure S3) and HSQC (Figure S4) spectra displayed ten tertiary sp^2^ carbons *(*δ_C_ 172.4, 168.6, 151.5, 145.6, 144.8, 143.9, 143.7, 141.0, 126.6 and 112.2), ten aromatic sp^2^ carbons (δ_C_ 137.8, 136.6, 135.3, 134.7, 133.4, 131.5, 128.7, 128.3, 116.1 and 113.3), an oxygenated methine carbon *(*δ_C_ 49.5) and a methyl carbon *(*δ_C_ 24.3) (Table 2). The characteristic UV spectrum (λ_max_ 256 and 371 nm), distinct deshielded aromatic proton signals at δ_H_ 8.90–7.98, an exchangeable proton signal for a carboxyl group at δ_H_ 15.60, a downshifted methine proton signal at δ_H_ 6.06 and four nitrogen-bearing quaternary carbons at δ_C_ 144.8, 143.9, 143.7 and 141.0 revealed the presence of a saphenic acid bearing phenazine moiety [7,8]. Analysis of the COSY spectrum (Figure S5) suggested four spin systems: From H-2 (δ_H_ 8.90) to H-4 (δ_H_ 8.70), from H-7 (δ_H_ 7.98) to H-9 (δ_H_ 8.23), from H-1’ at (δ_H_ 6.06) to H-2’ (δ_H_ 1.81) and from H-3” (δ_H_ 7.89) to H-6” (δ_H_ 6.42) (Figure 2). These evidences revealed that **1** had 1,6- or 1,9-disubstituted phenazine ring. Since ^1^H and ^13^C NMR chemical shift values of **1** were very similar to **4**, as well as already reported saphenic acid [9], the structure of **1** was concluded to be a 1,6-disubstituted phenazine derivative (Table 1 and Table 2). The molecular formula, based on the HR-MS result, revealed that **1** has an amino group. The amino group was linked to a remaining unconnected quaternary carbon C-7” by its chemical shift (δ_C_ 151.5). These data clearly indicated that **1** belongs to the saphenic acid family with 2-aminobenzoic acid moiety. The position of the carboxylic acid (1-COOH) was determined by the HMBC correlation between H-2 (δ_H_ 8.90) and the carbonyl carbon *(*δ_C_ 168.6) (Figure S6). The oxygenated methine proton H-1’ (δ_H_ 6.06) showed HMBC cross-peaks with C-5a (δ_C_ 143.7), C-6 (δ_C_ 145.6) and C-7 (δ_C_ 128.7). The HMBC correlation between the aromatic methine H-3” (δ_H_ 7.89) and C-1” (δ_C_ 172.4) established the presence of 2-amino benzoic ester (Figure 2). Though we could not directly ascertain the HMBC correlation between the saphenic acid moiety and 2-amino benzoic ester, the ROESY correlation between H-1’ (δ_H_ 6.06) and H-6” (δ_H_ 6.42) established their connection (Figure S7). Furthermore, saphenic acid and 2-amino benzoic ester satisfied the unsaturation number and molecular formula. Thereby, the oxygenated methine C-1’ was found to be connected with C-1” by an ester linkage. Thus, the structure of **1** was determined as 6-[1-(2-aminobenzoyloxy)ethyl]-1-phenazinecarboxylic acid.

**Figure 3:**
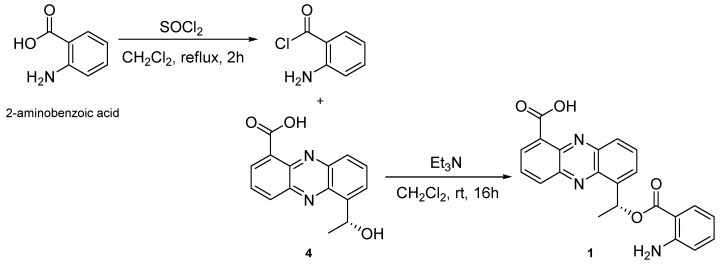
Semi-synthesis of **1**.

It was supposed that this strain produces phenazine derivatives through the same biosynthesis pathway [6]. So, we assumed that the stereocenter of C-1’ has the same configuration with (*R*)-saphenic acid (**4**). To determine the stereochemistry of C-1’ in **1**, **1** was semi-synthesized with **4** and 2-aminobenzoic acid (Figure 3). Semi-synthesized **1** showed identical ^1^H NMR and MS spectra with natural **1** (Figures S26–S28). In addition, natural and semi-synthesized **1** had the same negative optical rotation values (*R*-saphenic acid: [α]${}_{D}^{25}$ −73, (*c* 0.05, CHCl_3_); natural **1**: [α]${}_{D}^{25}$ −80, (*c* 0.05, CHCl_3_); semi-synthetic **1**: [α]${}_{D}^{25}$ −40, (*c* 0.05, CHCl_3_)). Thus, the stereochemistry of C-1’ was determined to be *R*-configuration. Peculiarly, **1** was easily changed under the light. However, it could be changelessly stored in the dark and fridge for several weeks.

Compound **2** was purified as a yellowish needle. The molecular formula of **2** was deduced to be C_15_H_13_N_3_O_2_ with 11 degrees of unsaturation from the [M + H]^+^ peak at *m*/*z* 268.1088 (calcd. for 268.1086) in the HRESIMS. Based on the HRMS data (Figure S8), **2** had one more nitrogen and one less oxygen than saphenic acid (**4**). Chemical shifts and splitting patterns of **2** (Figures S9 and S10) were closely similar to those of **4**. The main difference was the appearance of two exchangeable proton signals at δ_H_ 10.69 and δ_H_ 6.29 for amide protons in **2** instead of the exchangeable proton signal at δ_H_ 15.44 of a carboxyl group in **1**. The amide protons showed correlations each other in the COSY spectrum (Figure S12). Therefore, the planer structure of **2** was elucidated as a new derivative of saphenic acid and named as saphenic amide. **2** also had a negative optical rotation value [α]${}_{D}^{25}$ −13, (*c* 0.1, CHCl_3_), which was in agreement with that of **4** [α]${}_{D}^{25}$ −13 (*c* 0.1, CHCl_3_), suggesting that the stereochemistry of C-1’ was *R*-form.

Compound **3** was obtained as a yellowish amorphous solid. Based on the HRESIMS spectrum (Figure S20), the molecular formula was established as C_16_H_16_N_2_O_2_ with 10 degrees of unsaturation from the [M + H]^+^ peak at *m*/*z* 269.1290 (calcd. for 269.1290). The ^1^H NMR (Figure S15) and HSQC (Figure S17) spectra of **3** showed three aromatic proton signals at δ_H_ 8.19 (1H, d, *J* = 8.2 Hz), 7.83 (1H, t, *J* = 6.9 Hz) and 7.78 (1H, d, *J* = 6.9 Hz), an oxygenated methine proton signal at δ_H_ 5.71 (1H, q, *J* = 6.5 Hz) and a methyl signal at δ_H_ 1.81 (1H, d, *J* = 6.5 Hz). ^13^C NMR and HSQC spectra displayed three quaternary carbons (δ_C_ 142.8, 141.4 and 141.0), three aromatic sp^2^ carbons (δ_C_ 131.4, 128.6 and 127.7), an oxygenated methine carbon (δ_C_ 68.9) and a methyl carbon (δ_C_ 23.8). The pattern of ^1^H NMR spectrum and UV maximum at 255 and 366 nm revealed the presence of a typical phenazine moiety. The main difference between **3** and the other isolated compounds was the disappearance of a carboxyl group in **3**. ^1^H and ^13^C NMR data of **3** showed only a half set of signals and **3** had no optical activity, indicating that **3** consisted of two equivalent molecular portions and had a symmetric structure of 1-(2,3-diaminophenyl)ethanol. By considering NMR chemical shifts and biosynthetic pathway of **3** and **4**, the stereochemistry of C-1’ in **3** was determined to be the same as that in **4**. Thus, the structure of **3** was determined to be a new saphenic acid derivative and named as saphenol.

The structures of the known compounds **4**–**7** were identified as the previously reported (*R*)-saphenic acid (**4**), phenazine-1-carboxylic acid (**5**), 6-(1-hydroxyehtyl)phenazine-1-carboxylic acid (**6**) and 6-acetyl-phenazine-1-carboxylic acid (**7**) by the comparison of NMR, MS and optical rotation values with the literature [9,10]. When comparing with saphenic acid (**4**), **5** was a lack of 1-hydroxy-ethyl group, **6** was methylated form of saphenic acid (**4**), and **7** was oxidized saphenic acid. As previously reported [9], saphenic acid (**4**) of yellow needle form turned brown after several hours in day light as well as fluorescent light. Moreover, **4** was quickly turned from yellow into orange in hydrophilic solvents like methanol and dimethyl sulfoxide under the light within a few minutes.

The anti-inflammatory activity of **1**–**7** was assessed by NO assay, and their cytotoxicity on RAW 264.7 cells was evaluated by 2,3-bis-(2-methoxy-4-nitro-5-sulfophenyl)-2H-tetrazolium-5-carboxanilide (XTT) assay. The biological activities of the previously reported compounds **4**–**7** have never been reported [9]. Even though saphenic acid is a common pharmacophore for antibiotics and antitumor reagents, its biological activity has never been reported [11].

**Figure 4:**
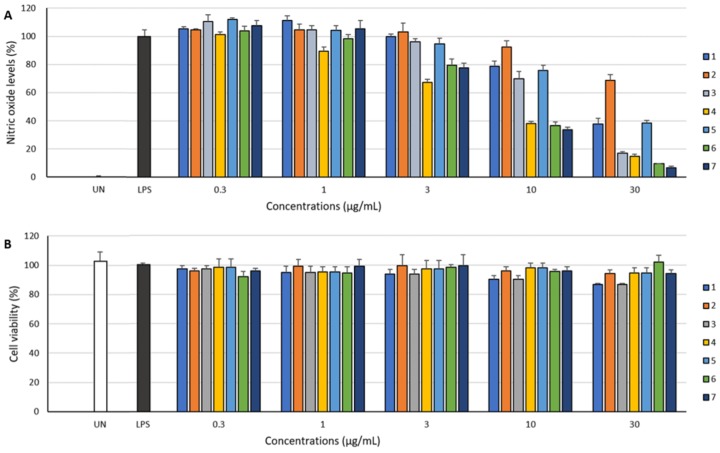
Effects of **1**–**7** on production of nitric oxide (NO) in LPS-induced macrophage RAW 264.7 cells (**A**) and cell viability of RAW 264.7 cells (**B**).

**1**–**7**, except for **2**, exhibited inhibitory effect against LPS-induced NO production in RAW 264.7 cells (Figure 4). Moreover, these compounds did not affect the viability of RAW 264.7 cells at concentrations up to 30 μg/mL (Figure 4). Among the isolated compounds, **6** inhibited NO production with the highest EC_50_ value of 19.6 μM. Saphenic acid (**4**) and their derivatives (**6** and **7**) showed a similar inhibition tendency (Table 3). Methylated (**6**) and oxidized (**7**) saphenic acids showed no significant difference in the activity. However, these compounds were about twice stronger than saphenic acid substituted with 2-amino benzoic acid (**1**, EC_50_ = 46.8 μM). Furthermore, when there is no functional group at C-6 of phenazine (**5**, EC_50_ = 76.1 μM), the activity was lowest. We could not carry out further biological activity tests due to insufficient amount. However, our results suggest that NO inhibitory activity is influenced by the carboxylic acid at C-1 as well as the presence of simple functional group at C-6 of phenazine ring. 

## 3. Materials and Methods

### 3.1. General Experimental Procedures

Optical rotation was measured on a Rudolph research analytical Autopol III S2 polarimeter (Rudolph Research Analytical, Hackettstown, NJ, USA). UV spectra were obtained on a Shimadzu UV-1650PC spectrophotometer (Shimadzu Corporation, Kyoto, Japan). IR spectra were recorded on a JASCO FT/IR-4100 spectrophotometer (JASCO Corporation, Tokyo, Japan). NMR spectra were collected on a Bruker AVANCE III 600 spectrometer (Bruker BioSpin GmbH, Rheinstetten, Germany) operating at 600 MHz (^1^H) and 150 MHz (^13^C). LRMS data were acquired on an Agilent 6100 single quadrupole mass spectrometer (Agilent Technologies, Santa Clara, CA, USA) with electron ionization (EI). HRESIMS was obtained with a Waters SYNPT G2 Q-TOF mass spectrometer (Waters Corporation, Milford, CT, USA) at Korea Basic Science Institute (KBSI) in Cheongju, Republic of Korea. HPLC was performed using a PrimeLine binary pump (Analytical Scientific Instruments, Inc., El Sobrante, CA, USA) with Shodex RI-101 refractive index detector (Shoko Scientific Co. Ltd., Yokohama, Japan) and S3210 variable UV detector (Schambeck SFD GmbH, Bad Honnef, Germany). Columns used for HPLC were YMC-Pack Silica (250 mm × 10 mm i.d., 5 µm), YMC ODS-A (250 mm × 10 mm i.d., 5 μm) and YMC-Triart C_18_ (250 mm × 10 mm i.d., 5 µm and 250 mm × 4.6 mm i.d., 5 µm). Silica gel 60 (Merck, 230–400 mesh) and RP-C_18_ silica gel (YMC-Gel ODS-A, 12 nm S-75 μm) were used for open column chromatography. Mass culture was carried out in a 100 L fermenter (Fermentec Corporation, Cheongju, Republic of Korea). All solvents used were either HPLC grade or distilled prior to use. 

### 3.2. Microbial Material

The strain IV17-028 was isolated from a deep-sea sediment sample collected using a multi-corer (MC) mounted on the R/V ISABU from the Indian Ocean (date: 9 August 2017, latitude: 008° 07.5121′ S, longtitude: 068° 06.6033′ E, depth: 4317 m). A total of 1 g of the heated sediment sample in the dry oven at 60 °C for 30 min, was spread onto the surface of Bennett (BN)’s agar plates (1% glucose, 0.2% tryptone, 0.1% yeast extract, 0.1% beef extract, 0.5% glycerol, 3.2% artificial sea salt and 1.8% agar, pH 7.02 before sterilization). The plates were incubated for 5 days at room temperature and then 20 days at 4 °C fridge. The resulting colony of dark green color was transferred and maintained on the BN agar plate. The colors of the colonies formed were beige to green on the BN agar medium. The strain was identified as *Cystobasidium laryngis* on the basis of 26S rRNA sequence analysis. The sequence of IV17-028 was submitted to GenBank under accession number MK131277. 

### 3.3. Fermentation and Isolation of Metabolites

The seed and production cultures were carried out in the BN broth medium (1% glucose, 0.2% tryptone, 0.1% yeast extract, 0.1% beef extract, 0.5% glycerol, 3.2% artificial sea salt, pH 7.02 before sterilization). The seed culture was performed in a 2 L baffled Erlenmeyer flask containing 600 mL of autoclaved medium at 28 °C for 5 days on a rotary shaker at 140 rpm. The seed culture broth was inoculated into a 100 L fermenter containing 60 L of the broth medium under the aseptic condition. The fermenter was operated at 28 °C, 50 rpm and airflow rate of 20 liter per minute (LPM) in the dark for 10 days. After cultivation, the broth was extracted with ethyl acetate (EtOAc, 60 L) twice. The EtOAc extract was evaporated to obtain crude extract (46.5 g). A portion of crude extract (12.8 g) was partitioned with 85% methanol (MeOH) in H_2_O and hexane (Hex). 85% MeOH layer (1.72 g) was subjected to ODS vacuum column chromatography followed by stepwise gradient elution with MeOH/H_2_O (1:4, 2:3, 3:2, 4:1 and 5:0, v/v) as eluent. Insoluble part (23.1 mg) containing **4** obtained from the 60% MeOH fraction was recrystallized from acetone to give a pure **4** (9.9 mg) as yellow needles. The 60% MeOH fraction was again applied to an ODS flash column chromatography with a MeOH/H_2_O solvent system (4:6, 5:5, 6:4, 7:3 and 8:2, v/v). The subfraction eluted with MeOH/H_2_O (6:4) was purified by a reversed-phase HPLC (YMC ODA-S, 250 × 10 mm i.d., 5 μm; 48% MeOH in H_2_O; flow rate: 2.0 mL/min; detector: RI) to yield crude **2** (2.3 mg, *t*_R_ 50 min). The crude **2** was recrystallized in MeOH to afford a pure **2** (1.1 mg) as yellow crystals. The 80% MeOH fraction was purified by a RP-HPLC (YMC ODS-A, 250 × 10 mm i.d., 5 μm; 28% MeCN in H_2_O containing 0.01% TFA; flow rate: 3.5 mL/min; detector: UV; wavelength: 254 nm) to give **3** (0.7 mg, *t*_R_ 30 min). The remaining crude extract (33.7 g) was suspended in H_2_O and sequentially partitioned with Hex, CHCl_3_, EtOAc and BuOH. The CHCl_3_ soluble fraction (5.94 g) was separated on silica gel flash column chromatography with MC/MeOH (10:0, 20:1, 10:1 and 5:1, v/v). The MC:MeOH (20:1) fraction was collected into nine fractions. Subfractions 3–5 (34.1 mg) from the MC:MeOH (20:1) fraction was purified by a normal phase HPLC (YMC-Pack SIL; 250 × 10 mm i.d., 5 μm; EtOAc:Hex (2:8); flow rate: 2.0 mL/min; detector: RI) to yield **5** (0.7 mg, *t*_R_ 35 min) and **6** (0.5 mg, *t*_R_ 25 min). Subfractions 6 and 7 (4.8 g) were again applied to an ODS-A vacuum column chromatography by stepwise gradient elution with MeOH/H_2_O (1:4, 2:3, 3:2, 4:1 and 5:0, v/v). Each fraction was divided into three subfractions. The third 80%MeOH fraction (468.5 mg) was separated with a normal phase HPLC (YMC-Pack SIL; 250 × 10 mm i.d., 5 μm; EtOAc:Hex:MeOH (1:1:0.02); flow rate: 2.0 mL/min; detector: RI) to afford **1** (1.7 mg, *t*_R_ 8 min), **4** (2.1 mg, *t*_R_ 14 min) and **7** (1.3 mg, *t*_R_ 12 min). 

Compound **1**: Yellowish amorphous; [α]${}_{D}^{25}$ −13 (*c* 0.1, CHCl_3_); UV (CHCl_3_) λ_max_ (log ε) 371 (1.25), 256 (1.83) nm; IR (CHCl_3_) *ν*_max_ 3727, 3625, 2883, 1222, 770 cm^−1^; ^1^H and ^13^C NMR data (CD_3_OD), see Table 1 and Table 2; ^1^H NMR (CDCl_3_, 600 MHz) δ_H_ 15.60 (1H, s), 8.99 (1H, d, *J* = 6.7 Hz), 8.62 (1H, d, *J* = 8.6, 6.7 Hz), 8.15 (1H, d, *J* = 8.3 Hz), 8.05 (1H, t, *J* = 7.6 Hz), 7.98 (1H, d, *J* = 7.8 Hz), 7.95 (1H, d, *J* = 6.8 Hz), 7.91 (1H, t, *J* = 8.5 Hz), 7.10 (1H, t, *J* = 8.1 Hz), 6.56 (1H, t, *J* = 7.4 Hz), 6.39 (1H, d, *J* = 8.6 Hz), 6.03 (1H, q, *J* = 14.2, 7.6 Hz), 1.82 (3H, d, *J* = 6.7 Hz); HRESIMS *m*/*z* 388.1296 [M + H]^+^ (calcd. for C_22_H_18_N_3_O_4_, 388.1297).

Saphenic amide (**2**): Yellowish needle; [α]${}_{D}^{25}$ −13 (*c* 0.1, CHCl_3_); UV (CHCl_3_) λ_max_ (log ε) 370 (0.87), 255 (1.52) nm; IR (CHCl_3_) *ν*_max_ 3328, 2922, 1664, 1218, 770 cm^−1^; ^1^H and ^13^C NMR data (CDCl_3_), see Table 1 and Table 2; HRESIMS *m*/*z* 268.1088 [M + H]^+^ (calcd. for C_15_H_14_N_3_O_2_, 268.1086).

Saphenol (**3**): Yellowish amorphous; [α]${}_{D}^{25}$ 0 (*c* 0.1, CHCl_3_); UV (CHCl_3_) λ_max_ (log ε) 366 (0.61), 255 (1.41) nm; IR (CHCl_3_) *ν*_max_ 3413, 2957, 1218, 770 cm^−1^; ^1^H and ^13^C NMR data (CDCl_3_), see Table 1 and Table 2; HRESIMS *m*/*z* 269.1290 [M + H]^+^ (calcd. for C_16_H_17_N_2_O_2_, 269.1290).

Saphenic acid (**4**): Yellowish needle; [α]${}_{D}^{25}$ −13 (*c* 0.1, CHCl_3_); ^1^H and ^13^C NMR data (CDCl_3_), see Table 1 and Table 2; LREIMS *m*/*z* 269.1 [M + H]^+^.

Phenazine-1-carboxylic acid (**5**): Yellowish amorphous; ^1^H NMR (CDCl_3_, 600 MHz) δ_H_ 15.58 (1H, s), 8.97 (1H, d, *J* = 7.0 Hz), 8.52 (1H, d, *J* = 8.7 Hz), 8.34 (1H, d, *J* = 8.3 Hz), 8.28 (1H, d, *J* = 8.64 Hz), 8.04–7.96. (1H × 3, overlapped); LREIMS *m*/*z* 225.1 [M + H]^+^.

6-(1-hydroxyehtyl)phenazine-1-carboxylic acid (**6**): Yellowish amorphous; [α]${}_{D}^{25}$ −60° (*c* 0.05, CHCl_3_); ^1^H NMR (CDCl_3_, 600 MHz) δ_H_ 15.62 (1H, s), 8.95 (1H, d, *J* = 7.0 Hz), 8.53 (1H, d, *J* = 8.7 Hz), 8.17 (1H, d, *J* = 8.3 Hz) 8.05 (1H, br d), 8.03–8.00 (1H × 2, overlapped), 5.76 (1H, q, *J* = 12.9, 6.5 Hz), 3.42 (3H, s), 1.62 (3H, d, *J* = 6.4 Hz); LREIMS *m*/*z* 283.1 [M + H]^+^.

6-acetylphenazine-1-carboxylic acid (**7**): Yellowish amorphous; ^1^H NMR (CDCl_3_, 600 MHz) δ_H_ 15.26 (1H, s), 9.01 (1H, dd, *J* = 7.0, 1.3 Hz), 8.55 (1H, dd, *J* = 8.7, 1.3 Hz), 8.41 (1H, dd, *J* = 8.7, 1.3 Hz), 8.29 (1H, dd, *J* = 6.9, 1.3 Hz), 8.08 (1H, dd, *J* = 8.6, 7.1 Hz), 8.05 (1H, dd, *J* = 8.6, 6.9 Hz), 3.07 (3H, s); LREIMS *m*/*z* 267.1 [M + H]^+^.

### 3.4. Semi-Synthesis of ***1***

To a solution of 2-aminobenzoic acid (50.0 mg, 0.36 mM) in toluene (1.2 mL) was added thionyl chloride solution (1.8 mL, 1.8 mM) at room temperature and the mixture was refluxed for 3 h. After completion of the reaction was confirmed by TLC, the solvent was removed under vacuum to obtain the crude acid chloride as yellow oil, which was used for further reaction without purification [12]. 

To a solution of (*R*)-saphenic acid (**4**) (5.4 mg, 0.02 mM) in DCM (200 μL) was added triethylamine (5 μL) at 0 °C and stirred for 10 min. To this mixture, the solution of acid chloride (150 μL, 0.02 mM) in DCM (2.7 mL) was added drop wise at 0 °C and stirred at room temperature for 16 h. The reaction solution was evaporated in vacuo and separated on PTLC (Hex-EtOAc, 4:6). The fractions containing a yellowish spot (Rf 0.1 ~ 0.4) were combined and further purified by RP-HPLC (YMC Triart-C_18_, 250 × 4.6 mm i.d., 5 μm; 45% MeCN in H_2_O containing 0.01% TFA; flow rate: 1.0 mL/min; detector: RI) to give semi-synthesized **1** (0.2 mg, t_R_ 32 min). ^1^H NMR (CDCl_3_, 600 MHz) δ_H_ 15.61 (1H, s), 8.99 (1H, d, *J* = 5.8 Hz), 8.61 (1H, d, *J* = 7.5 Hz), 8.14 (1H, d, *J* = 8.6 Hz), 8.05 (1H, t, *J* = 7.26 Hz), 7.95 (1H, d, *J* = 7.8 Hz), 7.94 (1H, d, *J* = 5.5 Hz), 7.89 (1H, t, *J* = 8.5 Hz), 7.08 (1H, d, *J* = 7.1 Hz), 6.54 (1H, t, *J* = 7.4 Hz), 6.35 (1H, d, *J* = 8.6 Hz), 6.03 (1H, q, *J* = 12.7, 5.9 Hz), 1.80 (3H, d, *J* = 6.7 Hz) (Figure S26).

### 3.5. Nitrite Quantification

NO_2_^−^ accumulation was used as an indicator of NO production as described previously [13]. RAW 264.7 cells were plated at 5 × 10^5^ cells/mL, pre-treated with various concentrations (0.3, 1, 3, 10 or 30 μg/mL) of **1**–**7** and stimulated with LPS (200 ng/mL) for 24 h. The supernatants were mixed with an equal volume of Griess reagent (1% sulfanilamide, 0.1% naphthylethylenediamine dihydrochloride and 2% phosphoric acid) and incubated at room temperature for 10 min. NaNO_2_ was used to generate a standard curve, and the concentration of nitrite in the medium was determined by measuring optical density at 540 nm [13]. Cell viability was determined by XTT assay as described previously [14]. And it was confirmed that the concentration of compounds used in this study has no significant effect on cell viability.

## 4. Conclusions

Three new compounds (**1**–**3**) and four known compounds (**4**–**7**) of phenazine class were isolated from a rare marine yeast-like fungus *Cystobasidium laryngis*. The structures of the new compounds were elucidated by detailed spectroscopic data analysis. Moreover, the absolute stereochemistry of **1**–**4** and **6** was determined by comparison of optical rotation values with literature. All the isolated compounds **1**–**7**, except for **2**, exhibited moderate NO production inhibition activity without cytotoxicity. This is the first report on the isolation of the new and known phenazine derivatives from the yeast-like fungus *Cystobasidium laryngis* and their anti-inflammatory activity.

## 5. Patents

Shin, H.J.; Lee, H.-S.; Choi, B.-K.; Lee, H.-S.; Lee, Y.-J.; Lee, J. S.; Lee, J. Method of producing saphenic acid and its derivatives and use thereof. KR 10-2018-0167792, 21 December 2018.

## Figures and Tables

**Table 1:** ^1^H NMR (600 MHz) data of **1**–**4**.

Position	δ_H_, Mult. (*J* in Hz)											
	1 ^a^			2 ^b^			3 ^b^			4 ^b^		
2	8.90	d	(7.4)	9.05	d	(7.4)				8.94	d	(7.9)
3	8.15	t	(7.4)	8.02	t	(7.4)				8.02	t	(7.9)
4	8.70	d	(8.6)	8.45	d	(8.6)				8.48	d	(9.2)
7	7.98	ovl ^c^		7.87	d	(7.1)	7.78	d	(6.9)	7.94	ovl ^c^	
8	7.99	ovl ^c^		7.92	t	(7.1)	7.83	t	(6.9)	7.96	ovl ^c^	
9	8.23	d	(7.7)	8.17	d	(8.5)	8.19	d	(8.2)	8.14	d	(8.4)
1’	6.06	q	(6.4)	5.78	q	(6.6)	5.71	q	(6.5)	5.82	q	(6.5)
2’	1.81	d	(6.4)	1.84	d	(6.6)	1.81	d	(6.5)	1.78	d	(6.5)
3”	7.89	d	(7.6)									
4”	6.49	t	(7.6)									
5”	7.03	t	(8.0)									
6”	6.42	d	(8.0)									
1-COOH	15.60 ^b^	s								15.44	s	
1-CONH_2_				10.69	br s							
				6.29	br s							

^a, b^ Spectra were recorded in CD_3_OD and CDCl_3_, respectively. ^c^ Signals were overlapped with other signals.

**Table 2:** ^13^C NMR (150 MHz) data of **1**–**4**.

Position	δ_C_, Type							
	1 ^a^		2 ^b^		3 ^b^		4 ^b^	
1	126.6 ^c,f^	C	129.2 ^d^	C			124.9 ^e^	C
2	137.8 ^f^	CH	136.3	CH			137.6	CH
3	131.5	CH	130.3	CH			130.6	CH
4	136.6 ^f^	CH	134.3	CH			135.1	CH
4a	143.9 ^c,f^	C	141.8 ^d^	C			141.8 ^e^	C
5a	143.7 ^f^	C	142.5	C	141.0	C	142.4	C
6	145.6	C	143.3	C	142.8	C	144.0	C
7	128.7	CH	127.4	CH	127.7	CH	127.9	CH
8	134.7 ^f^	CH	131.9	CH	131.4	CH	133.4	CH
9	128.3	CH	128.5	CH	128.6	CH	127.2	CH
9a	144.8 ^f^	C	143.1	C	141.4	C	140.4	C
10a	141.0	C	140.8	C			139.9	C
1’	49.5 ^g^	CH	68.8	CH	68.9	CH	68.0	CH
2’	24.3	CH_3_	23.9	CH_3_	23.8	CH_3_	24.1	CH_3_
1”	172.4 ^f^	C						
2”	112.2	C						
3”	133.4 ^f^	CH						
4”	116.1	CH						
5”	135.3	CH						
6”	113.3	CH						
7”	151.5	C						
1-COOH	168.6 ^f^	C					166.0	C
1-CONH_2_			166.5 ^f^	C				

^a, b^ Spectra were recorded in CD_3_OD and CDCl_3_, respectively. ^c–e^ Assignments interchangeable. ^f^ Chemical shifts were obtained from the HMBC spectrum. ^g^ Chemical shift was overlapped with solvent signals.

**Table 3:** Effective Concentrations (EC_50_, μg/mL and μM) of **1**–**7** against NO assay.

		Compounds						
		1	2	3	4	5	6	7
EC_50_	(μg/mL)	18.10	>30	14.67	6.15	17.06	5.53	5.32
	(μM)	46.8	-	54.7	22.9	76.1	19.6	20.0

## Supplementary Materials

The followings are available online at https://www.mdpi.com/1660-3397/17/8/482/s1, Figure S1: ^1^H NMR spectrum of compound **1** (600 MHz, CD_3_OD), Figure S2: ^1^H NMR spectrum of compound **1** (600 MHz, CDCl_3_), Figure S3: ^13^C NMR spectrum of compound **1** (150 MHz, CD_3_OD), Figure S4: HSQC spectrum of compound **1** in CD_3_OD, Figure S5: COSY spectrum of compound **1** in CD_3_OD, Figure S6: HMBC spectrum of compound **1** in CD_3_OD, Figure S7: ROESY spectrum of compound **1** in CD_3_OD, Figure S8: HRESI-MS spectrum of compound **1**, Figure S9: ^1^H NMR spectrum of compound **2** (600 MHz, CDCl_3_), Figure S10: ^13^C NMR spectrum of compound **2** (150 MHz, CDCl_3_), Figure S11: HSQC spectrum of compound **2** in CDCl_3_, Figure S12: COSY spectrum of compound **2** in CDCl_3_, Figure S13: HMBC spectrum of compound **2** in CDCl_3_, Figure S14: HRESI-MS spectrum of compound **2**, Figure S15: ^1^H NMR spectrum of compound **3** (600 MHz, CDCl_3_), Figure S16: ^13^C NMR spectrum of compound **3** (150 MHz, CDCl_3_), Figure S17: HSQC spectrum of compound **3** in CDCl_3_, Figure S18: COSY spectrum of compound **3** in CDCl_3_, Figure S19: HMBC spectrum of compound **3** in CDCl_3_, Figure S20: HRESI-MS spectrum of compound **3**, Figure S21: ^1^H NMR spectrum of compound **4** (600 MHz, CDCl_3_), Figure S22: ^13^C NMR spectrum of compound **4** (150 MHz, CDCl_3_), Figure S23: ^1^H NMR spectrum of compound **5** (600 MHz, CDCl_3_), Figure S24: ^1^H NMR spectrum of compound **6** (600 MHz, CDCl_3_), Figure S25: ^1^H NMR spectrum of compound **7** (600 MHz, CDCl_3_), Figure S26: ^1^H NMR spectrum of semi-synthesized **1** (600 MHz, CDCl_3_), Figure S27: LR-MS spectrum of semi-synthesized **1**, Figure S28: Comparison of ^1^H NMR data between semi-synthesized **1** and natural **1**.
