# Supplementary material for: Phenazine Derivatives with Anti-Inflammatory Activity from the Deep-Sea Sediment-Derived Yeast-Like Fungus Cystobasidium laryngis IV17-028
Source: Mar Drugs. 2019 Aug 19;17(8):482. doi: 10.3390/md17080482 (PMC6722648; doi:10.3390/md17080482)
Supplement: Supplementary file 1 [file marinedrugs-17-00482-s001.pdf]

Supplementary Information

Phenazine Derivatives with Anti-inflammatory Activity from the Deep-Sea Sediment-Derived Yeast-Like Fungus *Cystobasidium laryngis* IV17-028

Hwa-Sun Lee [1], Jong soon Kang [2], Byeoung-Kyu Choi [1], Hyi-Seung Lee [1], Yeon-Ju Lee [1], Jihoon Lee [1] and Hee Jae Shin [1,*]

[1] Marine Natural Products Chemistry Laboratory, Korea Institute of Ocean Science Technology, 385 Haeyang-ro, Yeongdo-gu, Busan 49111, Republic of Korea; hwasunlee@kiost.ac.kr (H.-S.L.); choibk4404@kiost.ac.kr (B.-K.C.); hslee@kiost.ac.kr (H.-S.L.); yjlee@kiost.ac.kr (Y.-J.L.); jihoonlee@kiost.ac.kr (J.L.)

[2] Laboratory Animal Resource Center, Korea Research Institute of Bioscience and Biotechnology, 30 Yeongudangi-ro, Ochang-eup, Cheongwon-gu, Cheongju 28116, Republic of Korea; kanjon@kribb.re.kr

*Correspondence: shinhj@kiost.ac.kr; Tel.: +82-51-664-3341

Contents

Figure S1. ^{1}H NMR spectrum of compound **1** (600 MHz, CD_3OD).

Figure S2. ^{1}H NMR spectrum of compound **1** (600 MHz, $CDCl_3$).

Figure S3. ^{13}C NMR spectrum of compound **1** (150 MHz, CD_3OD).

Figure S4. HSQC spectrum of compound **1** in CD_3OD.

Figure S5. COSY spectrum of compound **1** in CD_3OD**.**

Figure S6. HMBC spectrum of compound **1** in CD_3OD.

Figure S7. ROESY spectrum of compound **1** in CD_3OD.

Figure S8. HRESI-MS spectrum of compound **1**.

Figure S9. ^{1}H NMR spectrum of compound **2** (600 MHz, $CDCl_3$).

Figure S10. ^{13}C NMR spectrum of compound **2** (150 MHz, $CDCl_3$).

Figure S11. HSQC spectrum of compound **2** in $CDCl_3$.

Figure S12. COSY spectrum of compound **2** in $CDCl_3$**.**

Figure S13. HMBC spectrum of compound **2** in $CDCl_3$.

Figure S14. HRESI-MS spectrum of compound **2**.

Figure S15. ^{1}H NMR spectrum of compound **3** (600 MHz, $CDCl_3$).

Figure S16. ^{13}C NMR spectrum of compound **3** (150 MHz, $CDCl_3$).

Figure S17. HSQC spectrum of compound **3** in $CDCl_3$.

Figure S18. COSY spectrum of compound **3** in $CDCl_3$**.**

Figure S19. HMBC spectrum of compound **3** in $CDCl_3$.

Figure S20. HRESI-MS spectrum of compound **3**.

Figure S21. ^{1}H NMR spectrum of compound **4** (600 MHz, $CDCl_3$).

Figure S22. ^{13}C NMR spectrum of compound **4** (150 MHz, $CDCl_3$).

Figure S23. ^{1}H NMR spectrum of compound **5** (600 MHz, $CDCl_3$).

Figure S24. ^{1}H NMR spectrum of compound **6** (600 MHz, $CDCl_3$).

Figure S25. ^{1}H NMR spectrum of compound **7** (600 MHz, $CDCl_3$).

Figure S26. ^{1}H NMR spectrum of semi-synthesized **1** (600 MHz, $CDCl_3$).

Figure S27. LR-MS spectrum of semi-synthesized **1**.

Figure S28. Comparison of ^{1}H NMR data between semi-synthesized **1** and natural **1**.

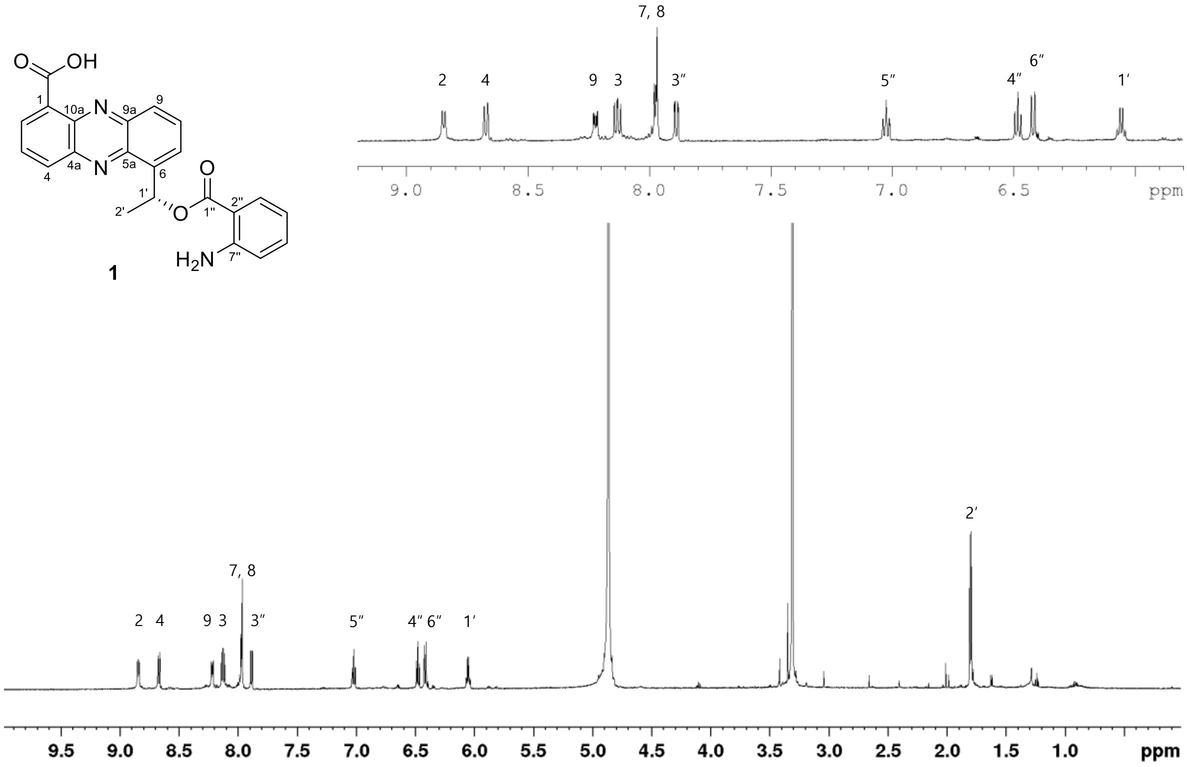

Figure S1. ^{1}H NMR spectrum of compound **1** (600 MHz, CD_3OD).

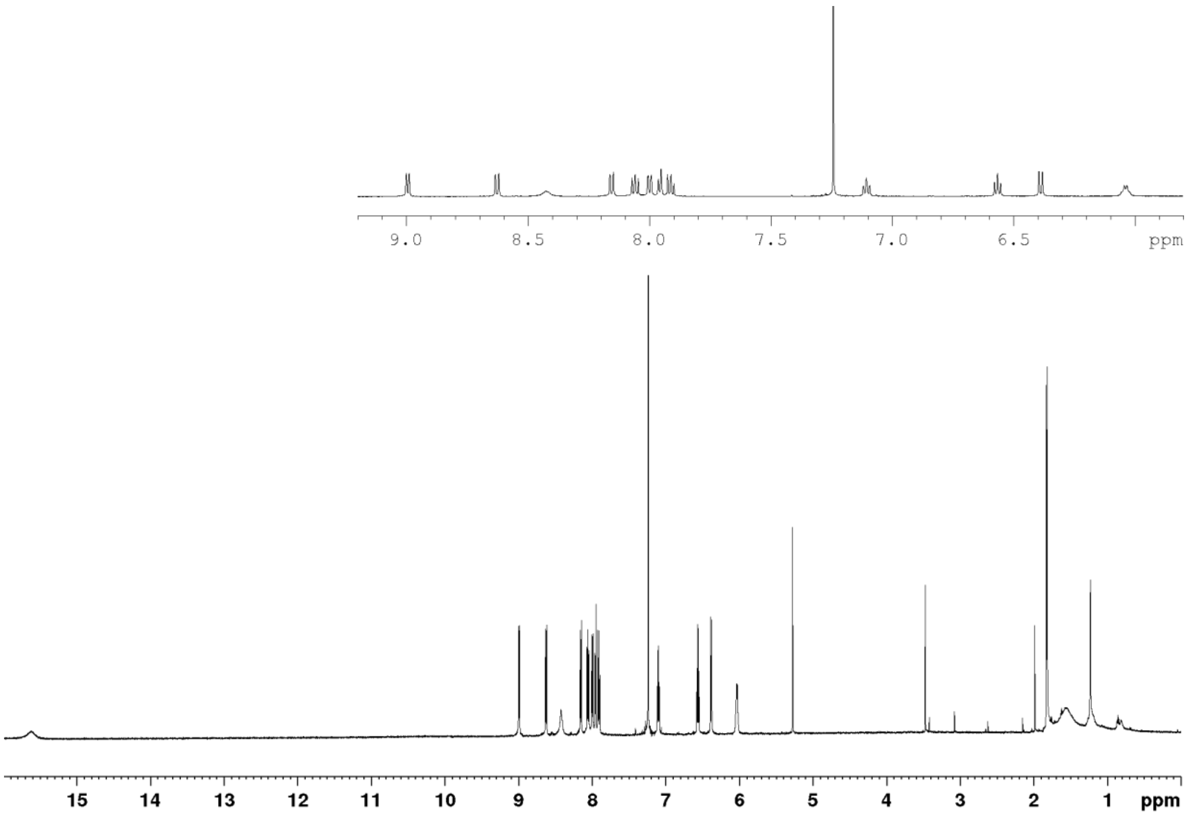

Figure S2. ^{1}H NMR spectrum of compound **1** (600 MHz, $CDCl_3$).

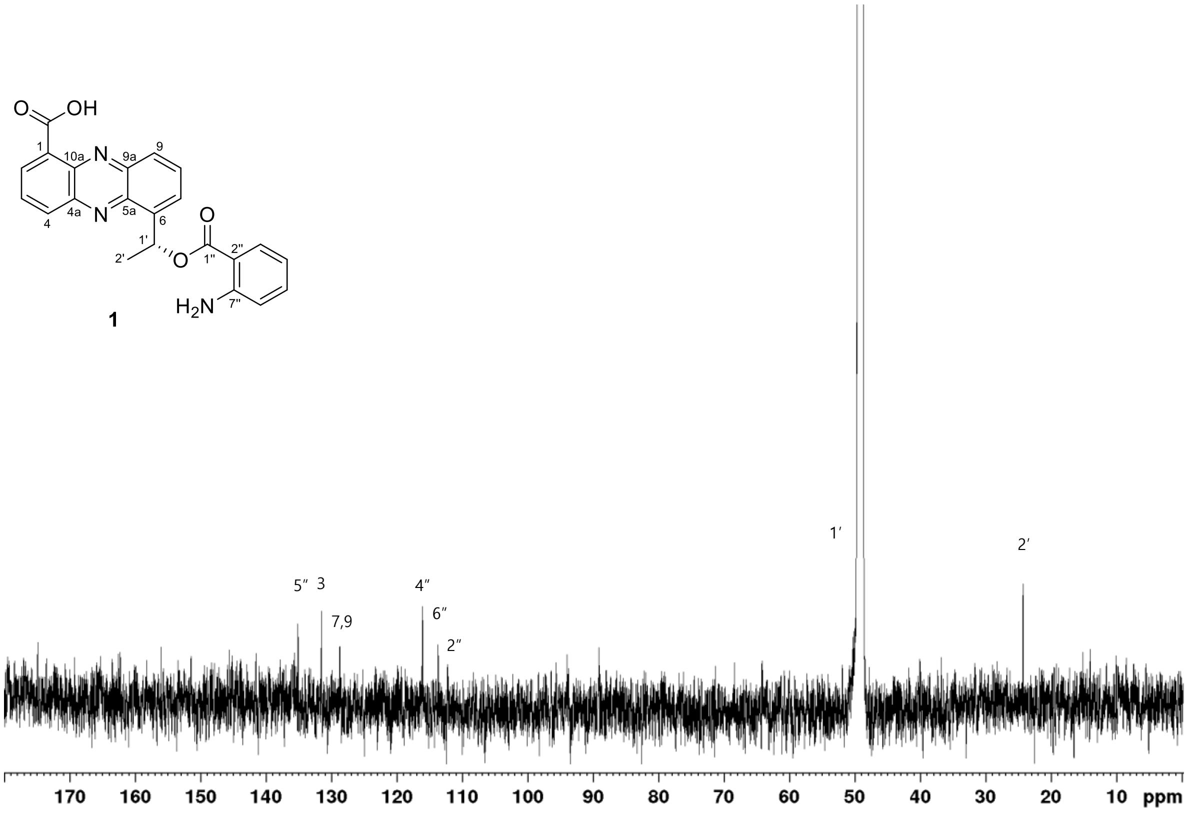

Figure S3. ^{13}C NMR spectrum of compound **1** (150 MHz, CD_3OD).

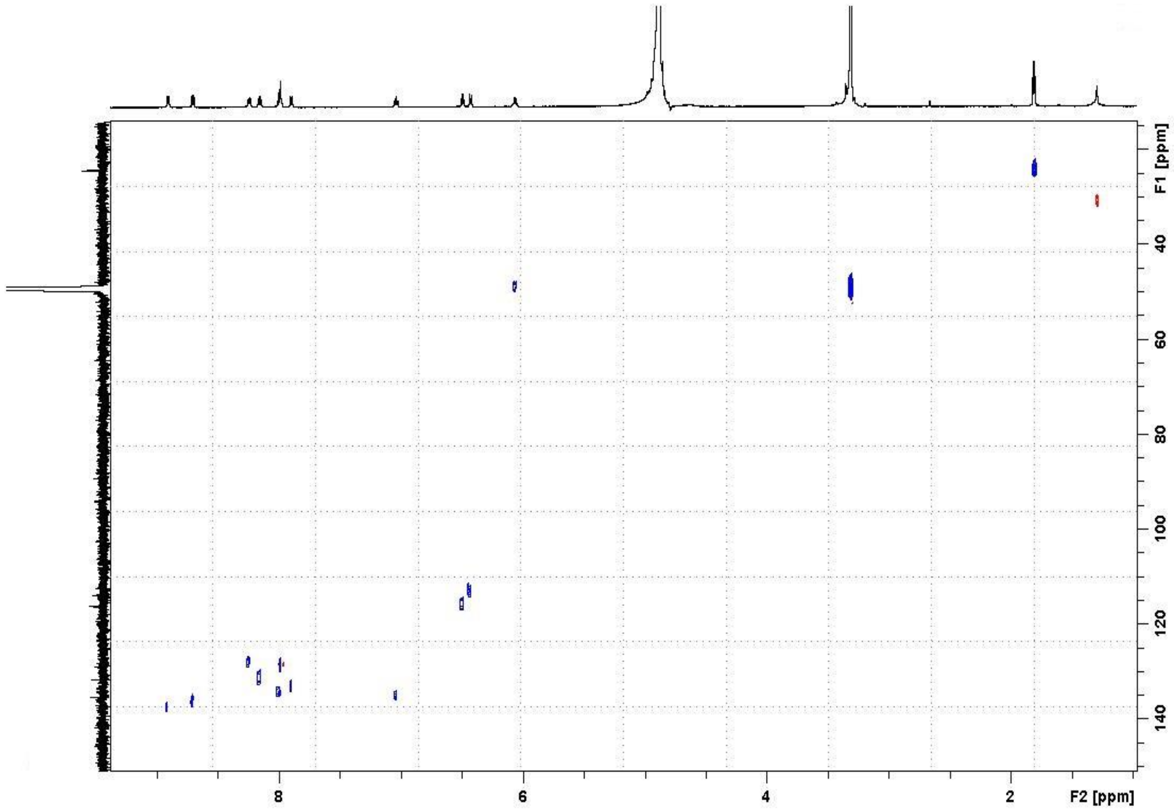

Figure S4. HSQC spectrum of compound **1** in CD_3OD.

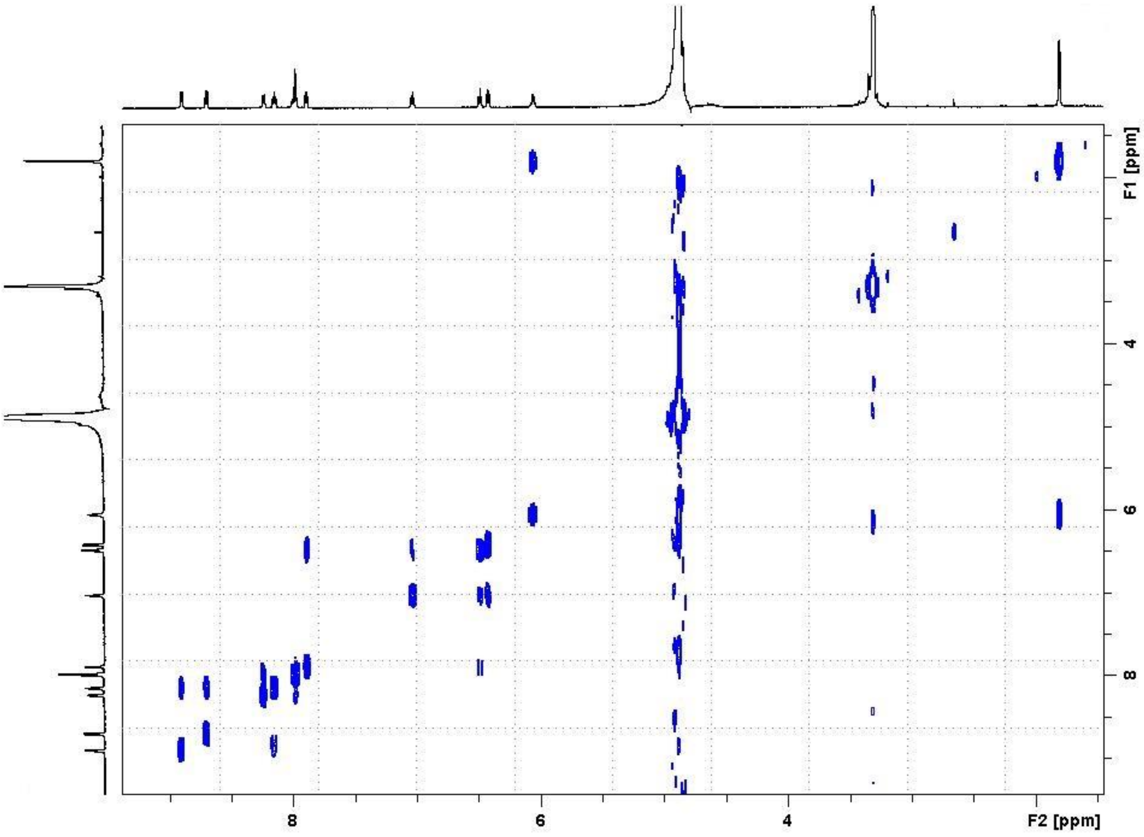

Figure S5. COSY spectrum of compound **1** in CD_3OD**.**

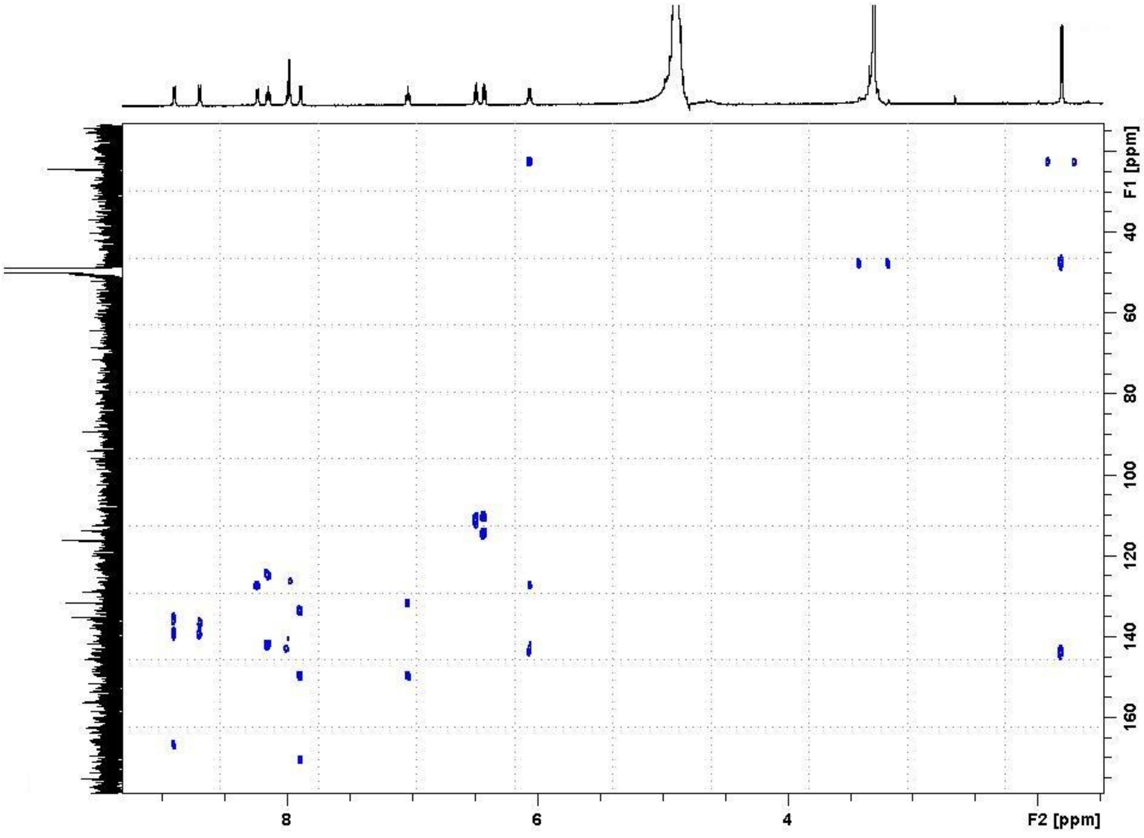

Figure S6. HMBC spectrum of compound **1** in CD_3OD.

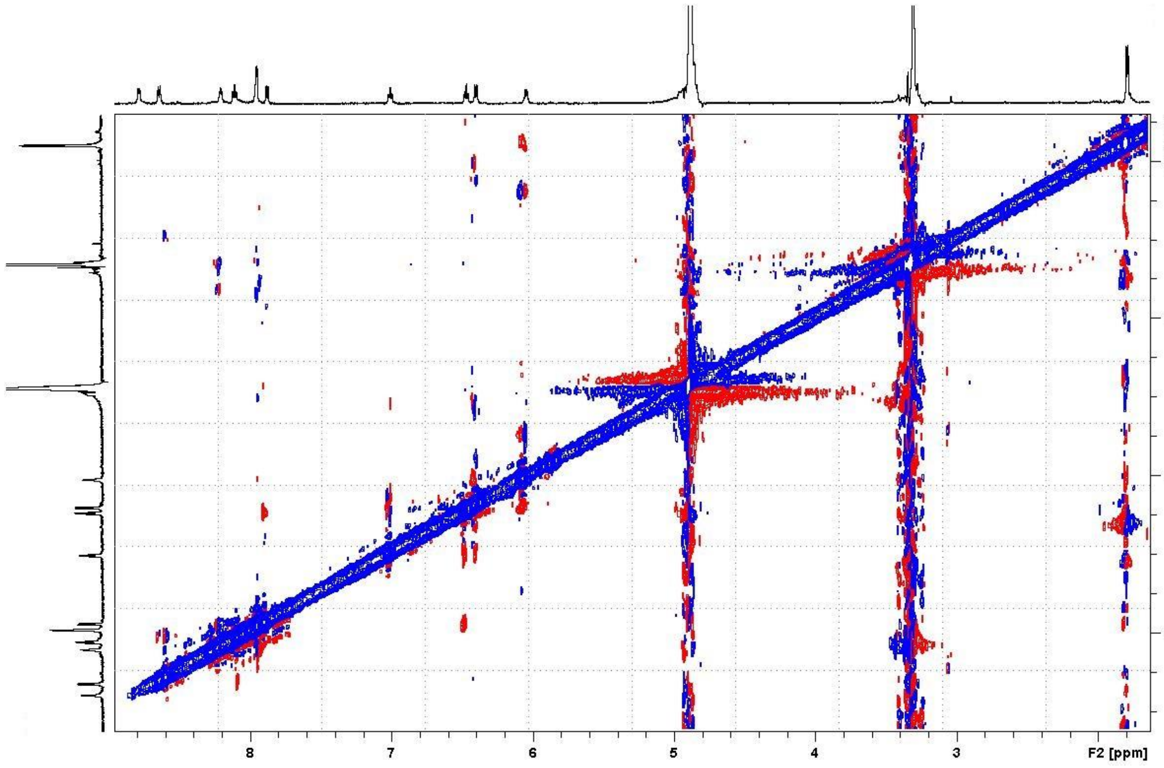

Figure S7. ROESY spectrum of compound **1** in CD_3OD.

Range : 365-435 m/z

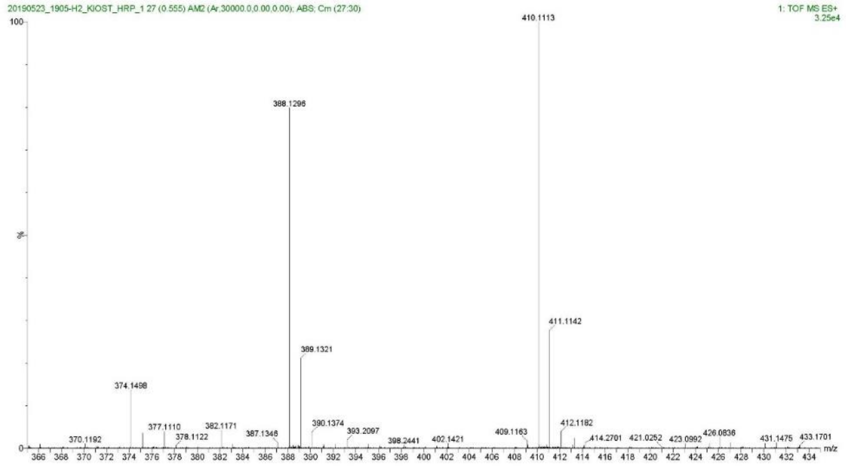

Elemental Composition Report

Single Mass Analysis
Tolerance = 5.0 PPM / DBE: min = -1.5, max = 100.0
Element prediction: Off
Number of isotope peaks used for i-FIT = 3

Monoisotopic Mass, Even Electron Ions
72 formula(e) evaluated with 2 results within limits (all results (up to 1000) for each mass)
Elements Used:
C: 0-25 H: 0-20 N: 1-3 O: 1-4 Na: 0-1

Minimum:				-1.5				
Maximum:		500.0	5.0	100.0				
Mass	Calc. Mass	mDa	PPM	DBE	i-FIT	Norm	Conf(%)	Formula
388.1296	388.1297	-0.1	-0.3	15.5	521.8	0.992	37.08	C22 H18 N3 O4
	388.1313	-1.7	-4.4	16.5	521.3	0.463	62.92	C25 H19 N O2 Na
410.1113	410.1117	-0.4	-1.0	15.5	609.9	n/a	n/a	C22 H17 N3 O4 Na

Figure S8. HRESI-MS spectrum of compound **1**.

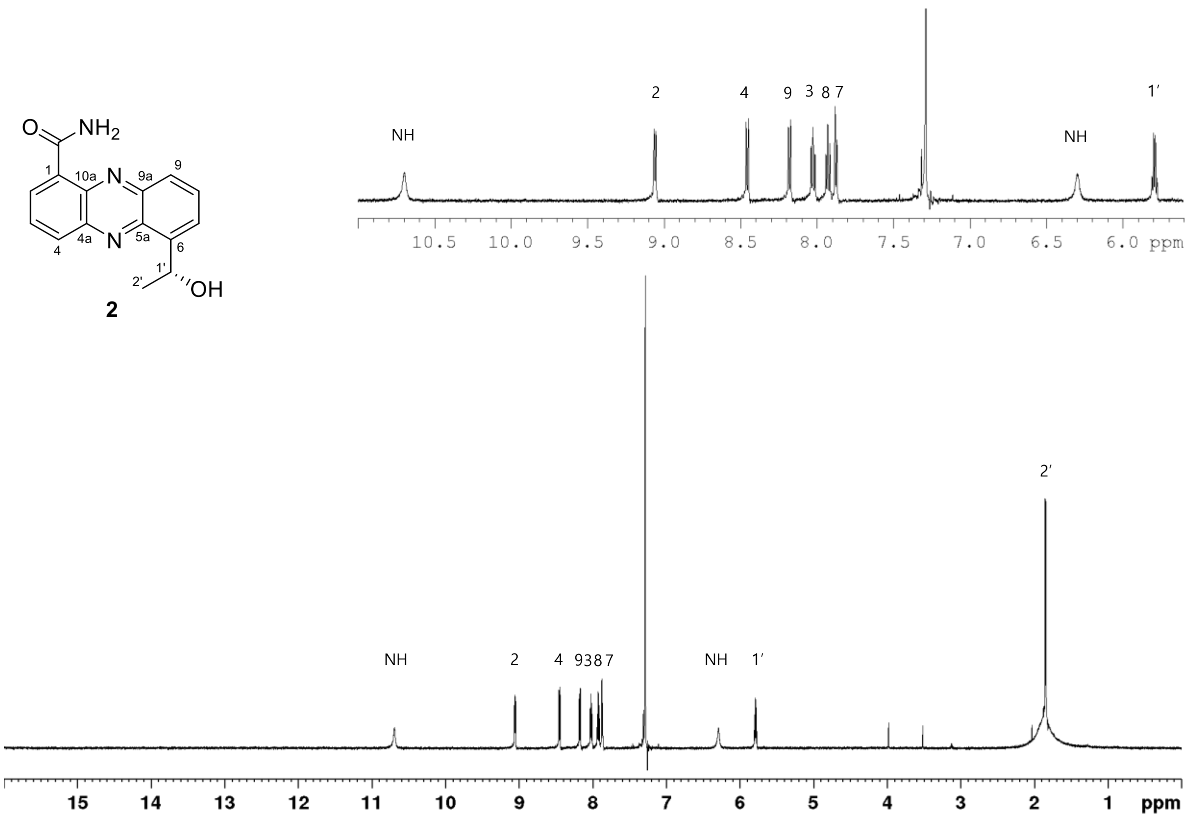

Figure S9. ^{1}H NMR spectrum of compound **2** (600 MHz, $CDCl_3$).

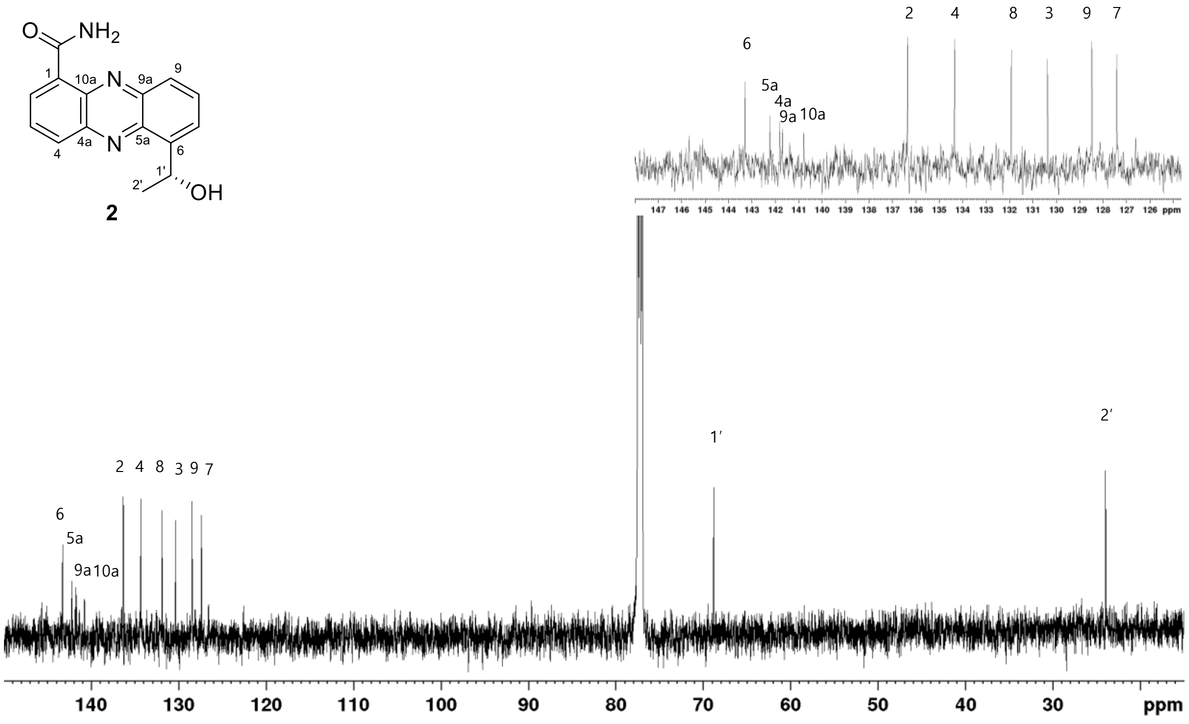

Figure S10. ^{13}C NMR spectrum of compound **2** (150 MHz, $CDCl_3$).

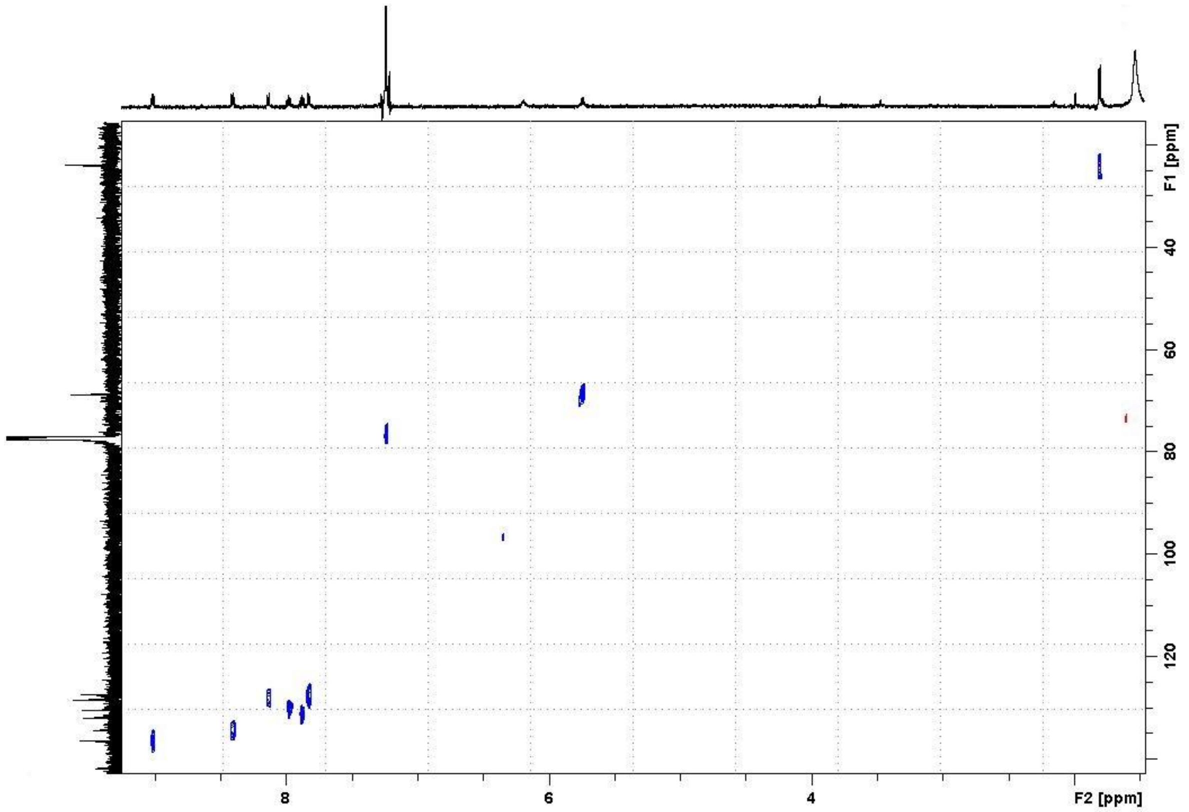

Figure S11. HSQC spectrum of compound **2** in $CDCl_3$.

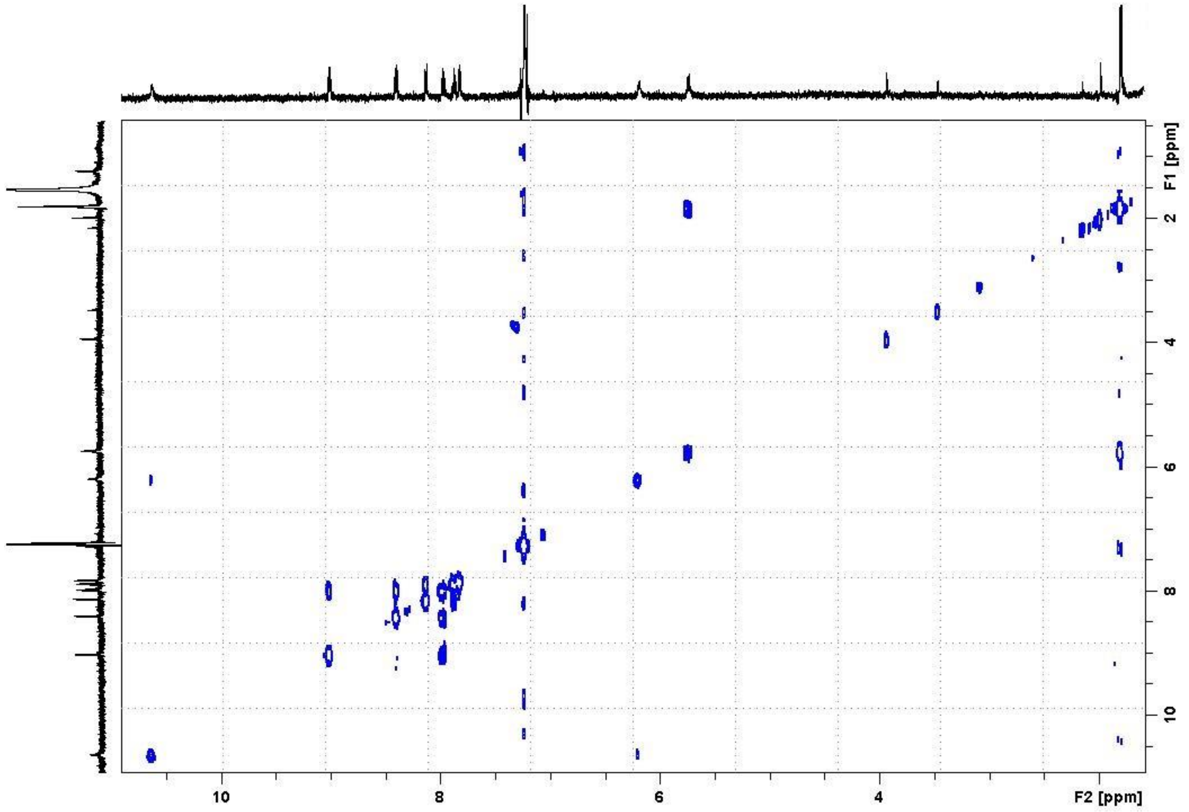

Figure S12. COSY spectrum of compound **2** in $CDCl_3$.

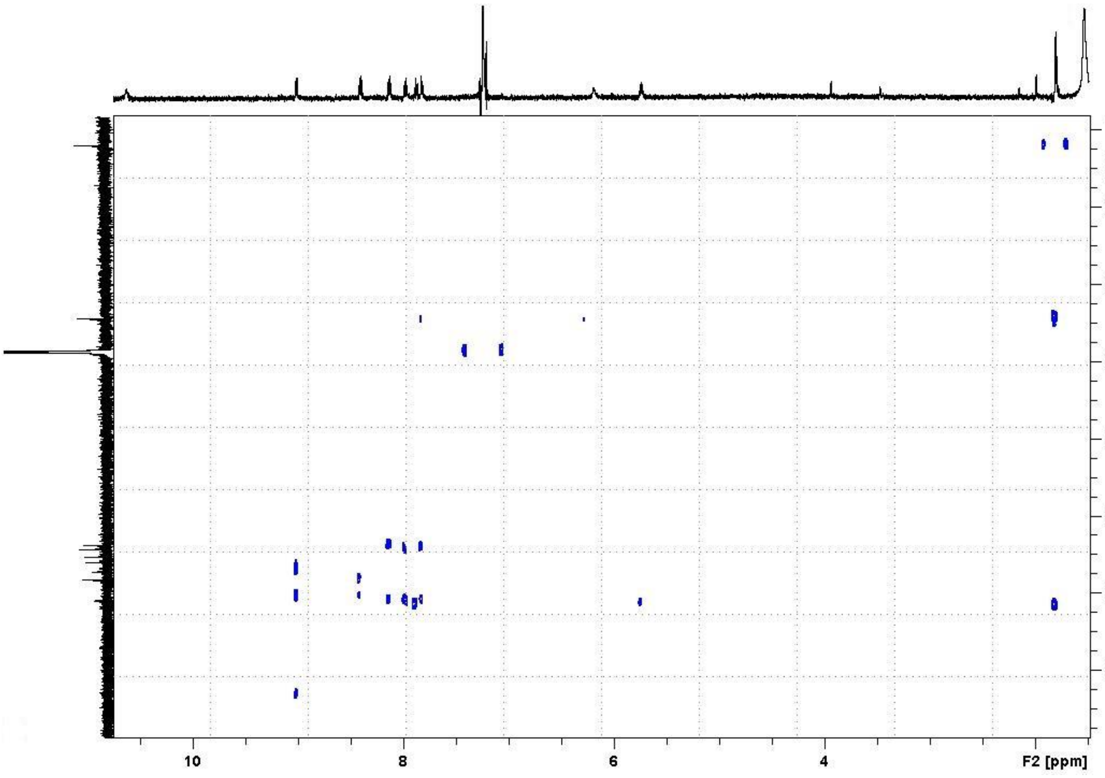

Figure S13. HMBC spectrum of compound **2** in $CDCl_3$.

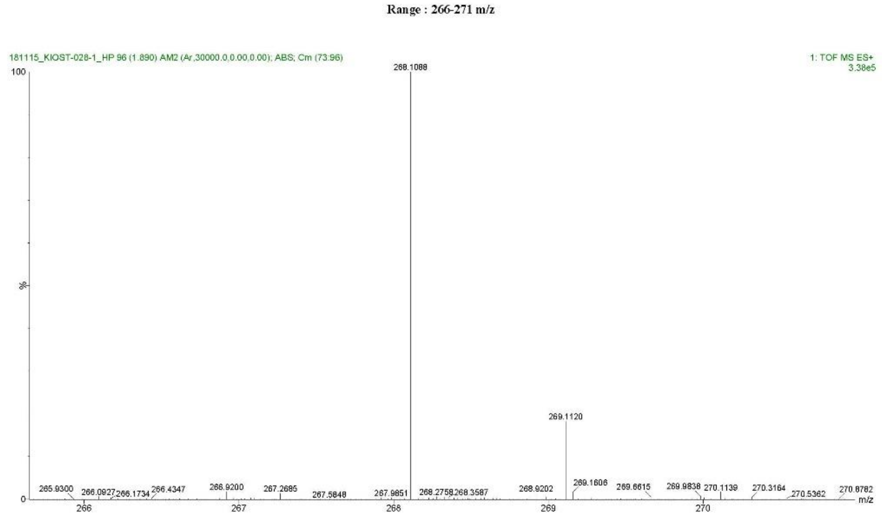

Elemental Composition Report

Single Mass Analysis
Tolerance = 50.0 PPM / DBE: min = -1.5, max = 50.0
Element prediction: Off
Number of isotope peaks used for i-FIT = 3

Monoisotopic Mass, Even Electron Ions
117 formula(e) evaluated with 2 results within limits (all results (up to 1000) for each mass)
Elements Used:
C: 15-40 H: 10-40 N: 1-10 O: 1-15

Minimum:				-1.5				
Maximum:		500.0	50.0	50.0				
Mass	Calc. Mass	mDa	PPM	DBE	i-FIT	Norm	Conf(%)	Formula
268.1088	268.1086	0.2	0.7	10.5	1146.3	0.013	98.73	C15 H14 N3 O2
	268.0974	11.4	42.5	10.5	1150.7	4.364	1.27	C16 H14 N O3

Figure S14. HRESI-MS spectrum of compound **2**.

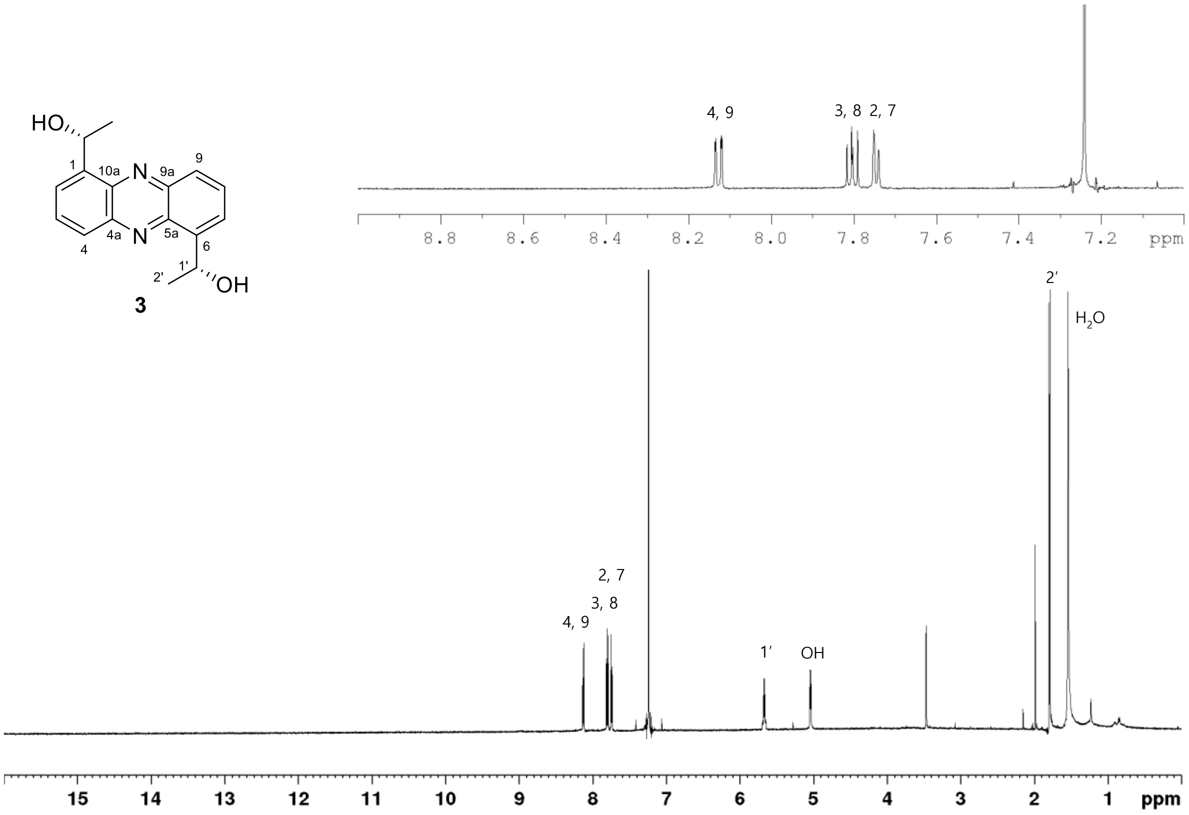

Figure S15. ^{1}H NMR spectrum of compound **3** (600 MHz, $CDCl_3$).

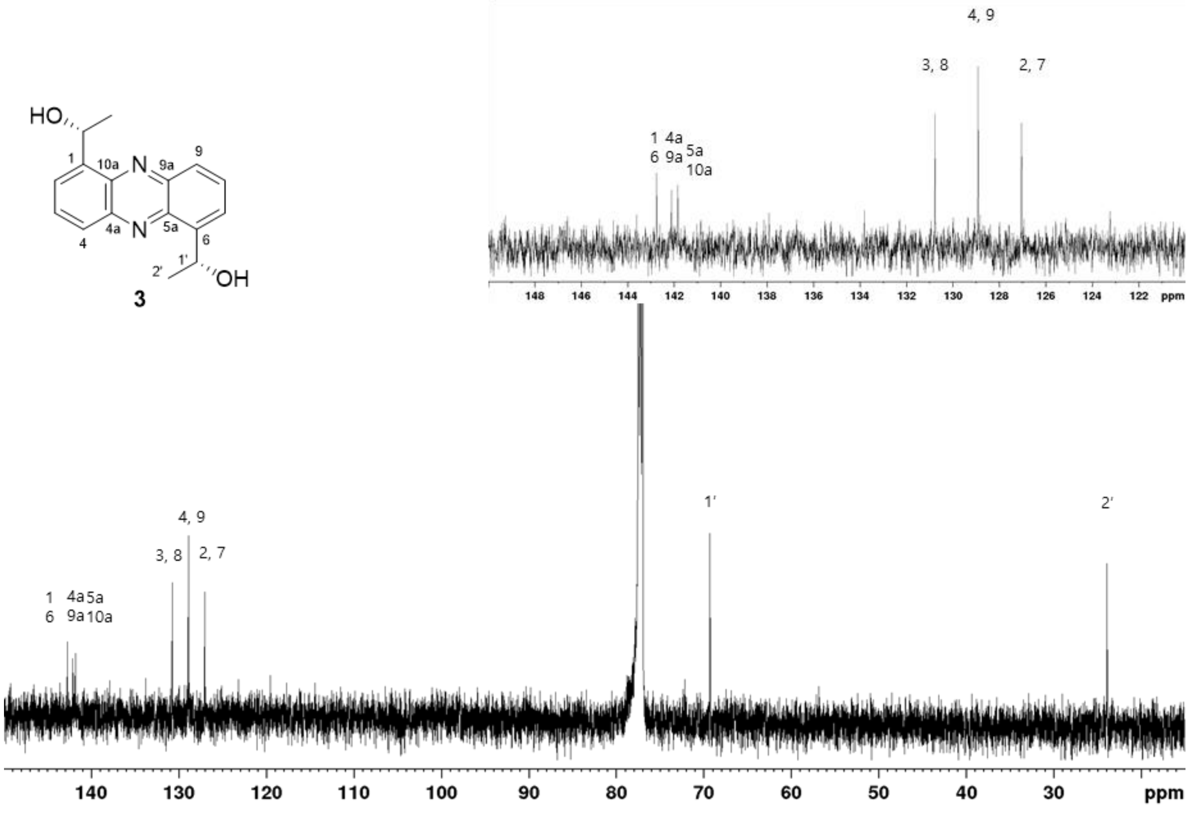

Figure S16. ^{13}C NMR spectrum of compound **3** (150 MHz, $CDCl_3$).

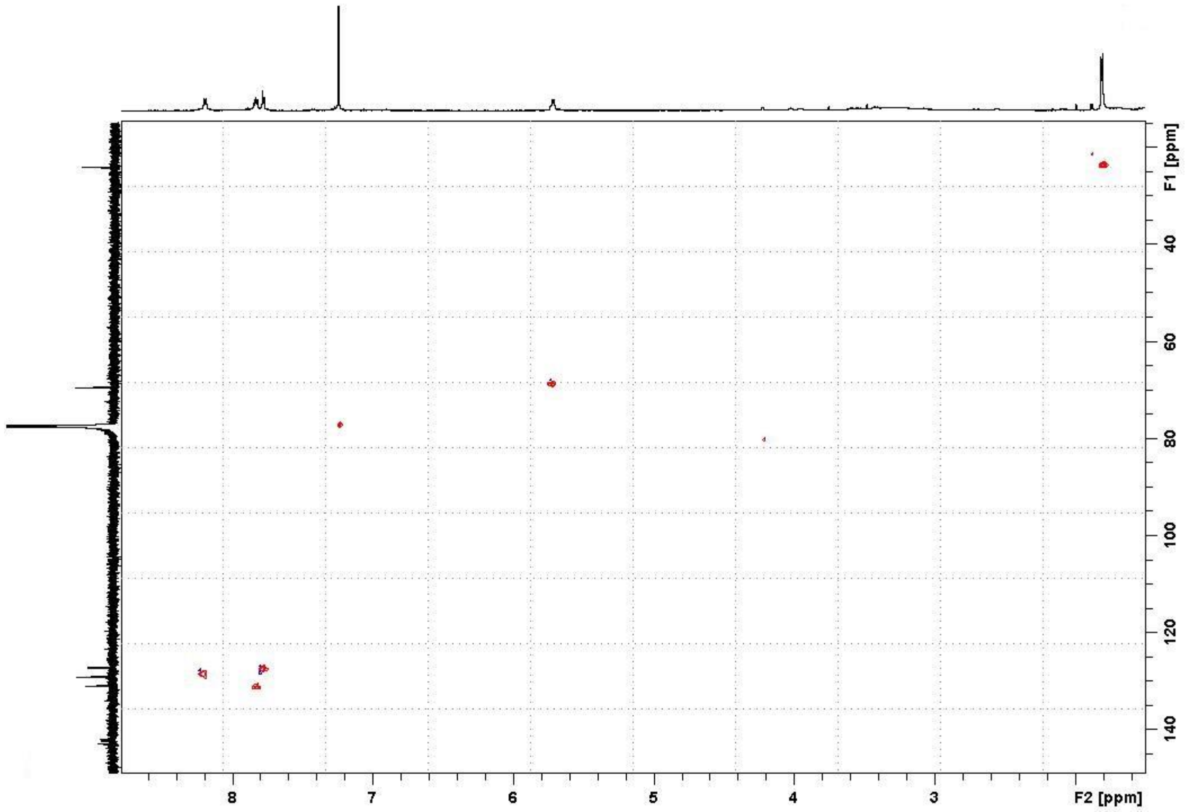

Figure S17. HSQC spectrum of compound **3** in $CDCl_3$.

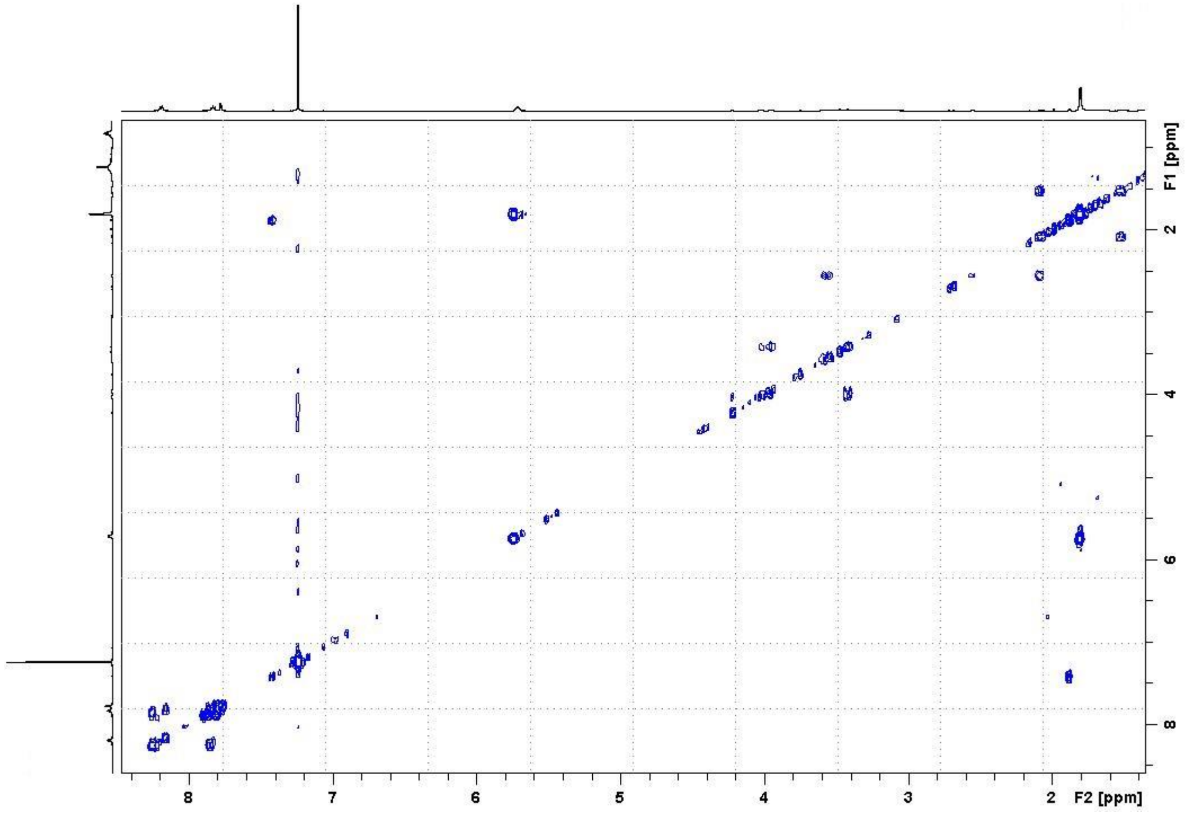

Figure S18. COSY spectrum of compound **3** in $CDCl_3$**.**

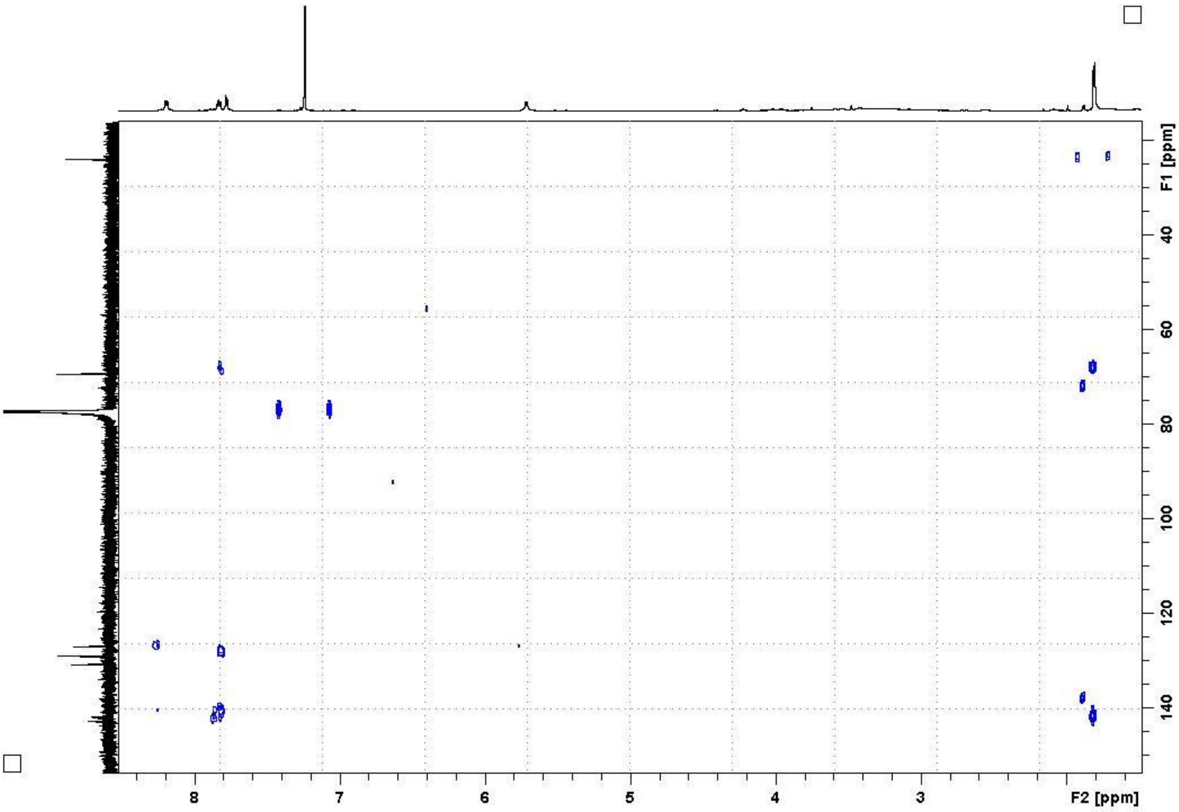

Figure S19. HMBC spectrum of compound **3** in $CDCl_3$.

Range : 255-310 m/z

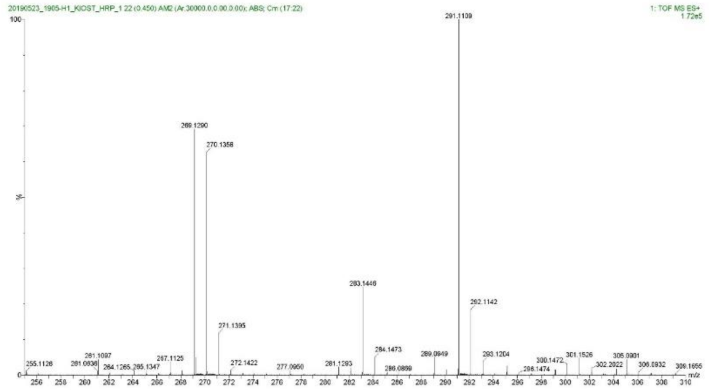

Elemental Composition Report

Single Mass Analysis
Tolerance = 5.0 PPM / DBE: min = -1.5, max = 100.0
Element prediction: Off
Number of isotope peaks used for i-FIT = 3

Monoisotopic Mass, Even Electron Ions
89 formula(e) evaluated with 1 results within limits (all results (up to 1000) for each mass)
Elements Used:
C: 0-25 H: 0-20 N: 1-3 O: 1-4 Mn: 0-1

Minimum:				-1.5				
Maximum:		500.0	5.0	100.0				
Mass	Calc. Mass	mDa	PPM	DBE	i-FIT	Norm	Conf(%)	Formula
269.1290	269.1290	0.0	0.0	9.5	1081.1	n/a	n/a	C16 H17 N2 O2
291.1109	291.1109	0.0	0.0	9.5	894.3	n/a	n/a	C16 H16 N2 O2 Na

Figure S20. HRESI-MS spectrum of compound **3**.

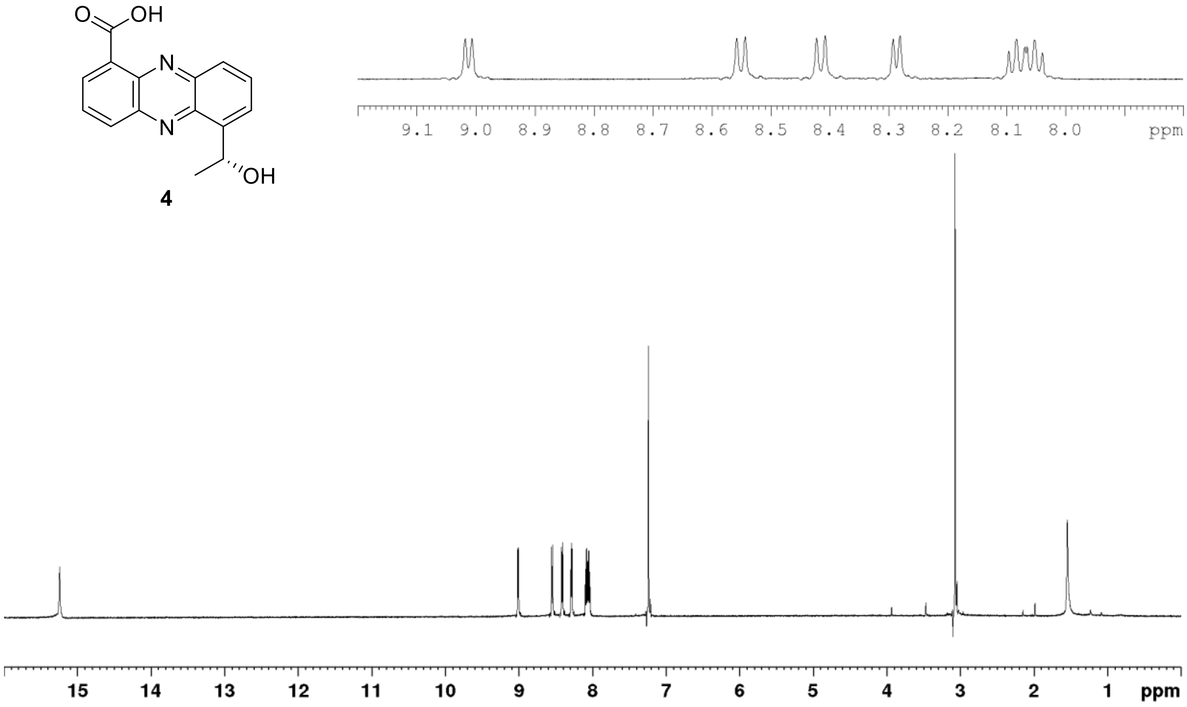

Figure S21. ^{1}H NMR spectrum of compound **4** (600 MHz, $CDCl_3$).

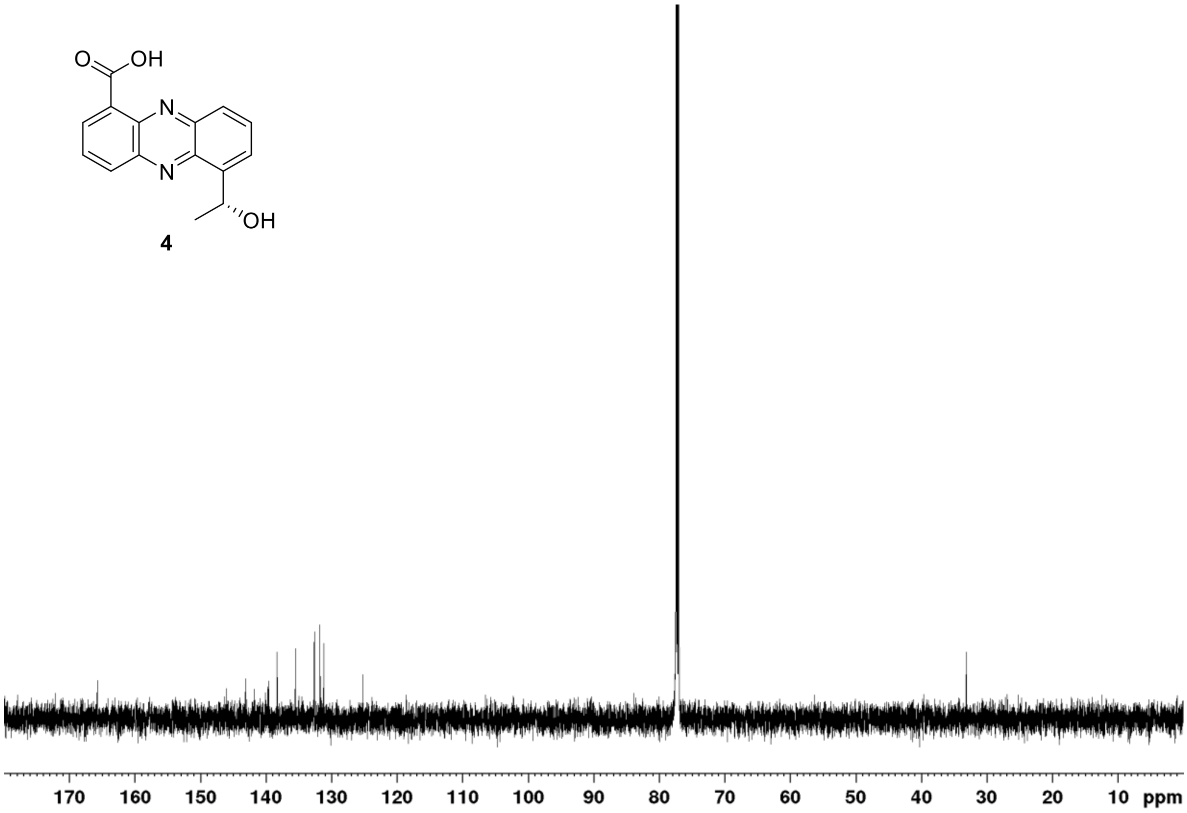

Figure S22. ^{13}C NMR spectrum of compound **4** (150 MHz, $CDCl_3$).

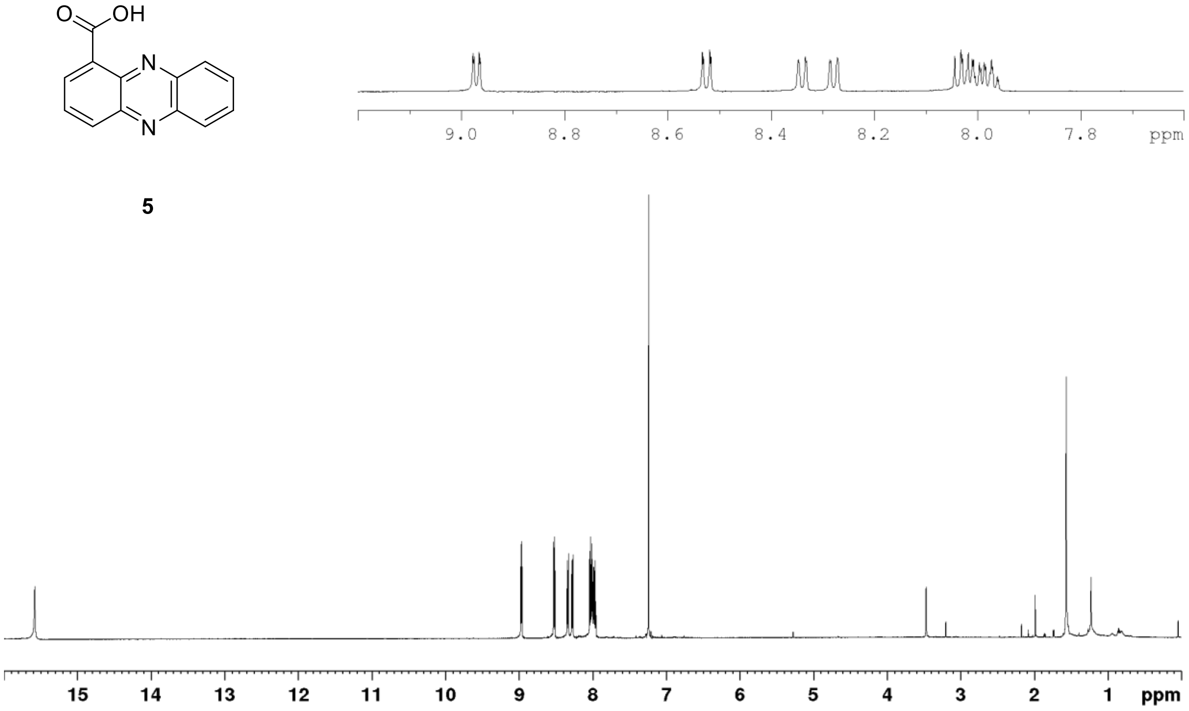

Figure S23. ^{1}H NMR spectrum of compound **5** (600 MHz, $CDCl_3$).

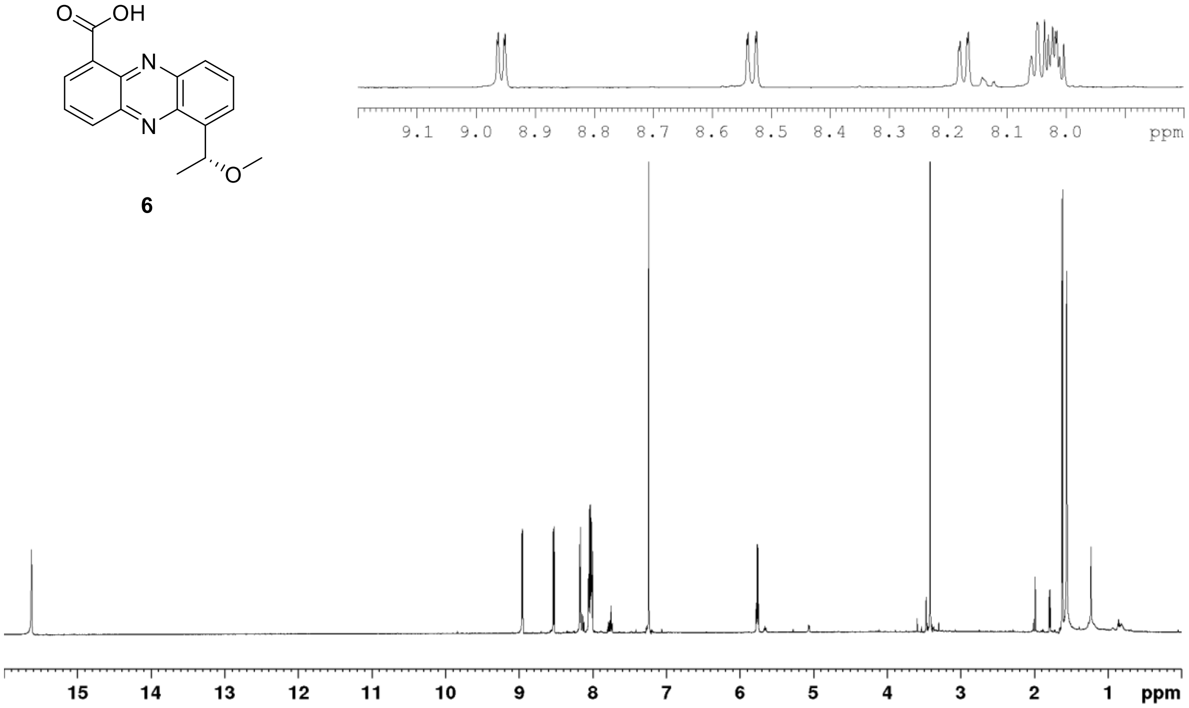

Figure S24. ^{1}H NMR spectrum of compound **6** (600 MHz, $CDCl_3$).

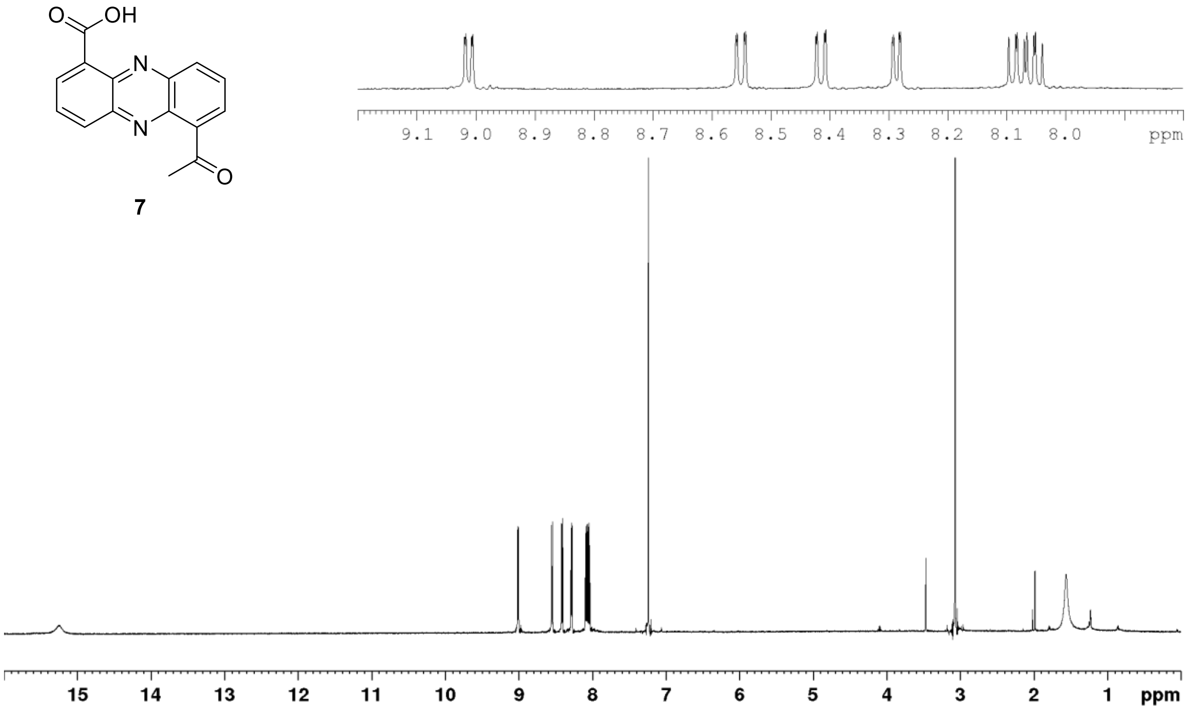

Figure S25. ^{1}H NMR spectrum of compound **7** (600 MHz, $CDCl_3$).

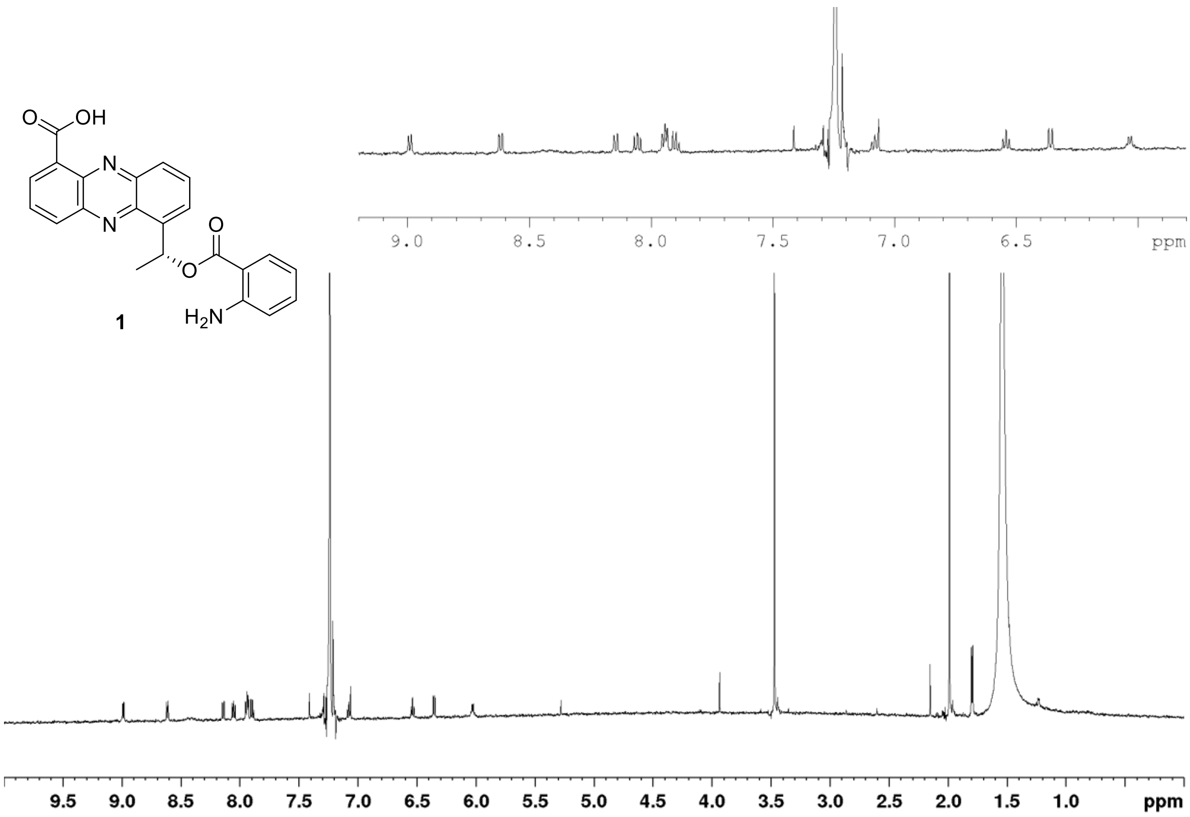

Figure S26. ^{1}H NMR spectrum of semi-synthesized **1** (600 MHz, $CDCl_3$).

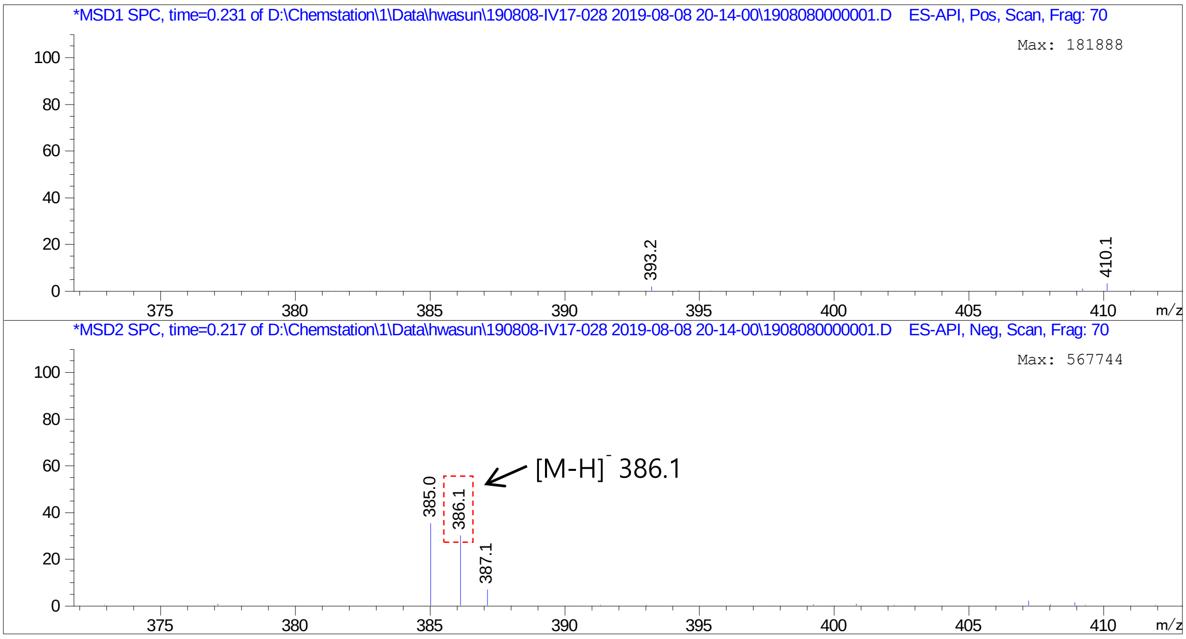

Figure S27. LR-MS spectrum of semi-synthesized **1**.

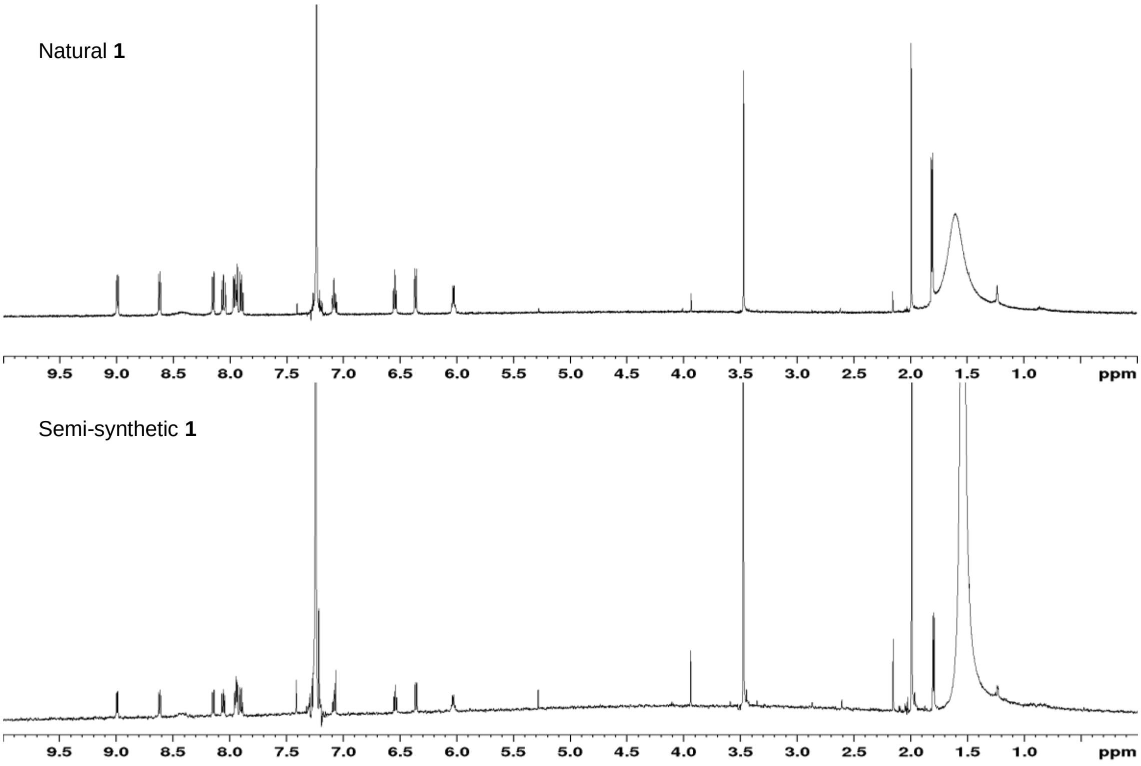

Figure S28. Comparison of ^{1}H NMR data between semi-synthesized **1** and natural **1**.
